# Tubule‐Derived IFN‐α Promotes GSDMD‐Mediated Macrophage Pyroptosis to Drive Renal Inflammation and Fibrosis Through JAK2/STAT2 Activation

**DOI:** 10.1002/advs.202512278

**Published:** 2025-12-12

**Authors:** Yiping Xu, Yating Wang, Siming Jiang, Yi Li, Guanglan Li, Yuchu Liu, Siyuan Li, Yiming Zhou, Qinghua Liu, Yi Zhou, Wei Chen, Hongyu Li, Haiping Mao

**Affiliations:** ^1^ Department of Nephrology The First Affiliated Hospital Sun Yat‐sen University Guangzhou 510080 China; ^2^ Department of Nephrology NHC Key Laboratory of ClinicalNephrology (Sun Yat-Sen University) and Guangdong Provincial Key Laboratory ofNephrology Guangzhou 510080 China; ^3^ Basic andTranslational Medical Research Center, Sun Yat-sen Memorial Hospital Sun Yat‐sen University Guangzhou 510120 China

**Keywords:** gasdermin D, inflammation, macrophages, pyroptosis, renal fibrosis

## Abstract

Macrophages exhibit high plasticity in response to tubular epithelial cell (TEC) injury. Gasdermin D (GSDMD)‐mediated pyroptosis amplifies the inflammatory and fibrogenic cascade, yet its role in chronic kidney disease (CKD) remains elusive. Herein, GSDMD is upregulated in kidney macrophages following unilateral renal ischemia‐reperfusion injury (UIRI) or folic acid‐induced injury, paralleling elevated pyroptosis rates. Clinically, the active fragment GSDMD‐N localizes primarily to CD68⁺ macrophages, and its renal level positively correlates with fibrosis severity across diverse CKD etiologies, reinforcing its pathogenic relevance. Macrophage‐specific deletion of *Gsdmd* ameliorates pyroptosis, inflammation, and renal fibrosis in both murine models, without affecting acute tubular damage in bilateral IRI. Mechanistically, injured TECs initiate this cascade through secreted IFN‐α, which activates the IFNAR1/JAK2/STAT2 axis in macrophages​. STAT2 then forms a complex with IRF9, directly binding to the *Gsdmd* promoter to transcriptionally upregulate GSDMD expression. Genetic ablation of *Jak2*, *Stat2*, or *Ifnar1* reduces GSDMD and GSDMD‐N levels and inhibits IL‐1β/IL‐18 secretion. Notably, administration of an IFN‐α neutralizing antibody attenuates UIRI‐induced pyroptotic macrophage, inflammation, and renal fibrosis. Collectively, the findings uncover a STAT2/IRF9‐dependent paracrine IFN‐α feedback loop that orchestrates GSDMD‐mediated pyroptosis, linking injured TECs to macrophage‐driven renal inflammation and fibrosis. Targeting this axis represents a promising strategy to halt CKD progression.

## Graphical abstract

Mao and colleagues uncover a STAT2/IRF9‐dependent signaling axis through which tubular epithelial cell (TEC)‐derived IFN‐α induces gasdermin D (GSDMD)‐mediated pyroptosis in macrophages. This TEC‐macrophage feedback loop amplifies renal inflammation and fibrosis, providing mechanistic insight into the progression of chronic kidney disease and revealing a potential direction for therapeutic intervention.

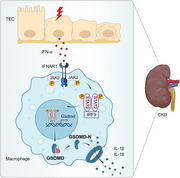

## 1 Introduction

Chronic Kidney Disease (CKD), a growing global public health issue affecting over 10% of the population,^[^ ^1^ ^]^ ultimately progresses to renal fibrosis irrespective of the underlying etiology. This terminal pathology features persistent sterile inflammation orchestrated by maladaptive tubular epithelial cells (TECs), which recruit inflammatory cells, activate myofibroblasts, and promote progressive extracellular matrix accumulation.^[^ ^2^, ^3^ ^]^ Current therapies lack efficacy in disrupting this inflammatory‐fibrotic cycle, rendering renal fibrosis irreversible and highlighting mechanistic gaps.

Macrophages are central regulators in renal inflammation and fibrogenesis.^[^ ^4^, ^5^ ^]^ In response to tubular injury, macrophages initially orchestrate tissue repair by limiting the injured TECs and promoting repair.^[^ ^6^, ^7^ ^]^ However, incomplete TEC resolution or persistent injury triggers macrophage activation, secreting inflammatory and profibrotic mediators that facilitate fibrosis.^[^ ^8^, ^9^ ^]^ Emerging evidence implicates pyroptosis, a gasdermin‐mediated cell death, as a critical modulator in this process. While macrophage pyroptosis protects against infections,^[^ ^10^, ^11^ ^]^ its dysregulation aggravates renal inflammation and fibrosis.^[^ ^12^, ^13^ ^]^ Notably, injured TECs induce macrophage pyroptosis through adenosine triphosphate (ATP) release, amplifying fibrotic responses in obstruction models.^[^ ^14^ ^]^ However, the precise signaling pathways linking TEC injury to macrophage pyroptosis remain poorly defined.

Gasdermin D (GSDMD), the key executor of pyroptosis, regulates plasma membrane pore formation and cytokine release.^[^ ^15^ ^]^ Canonical NOD‐like receptor family pyrin domain‐containing 3 (NLRP3) inflammasome activation begins with a priming signal, including pathogen‐associated molecular patterns (PAMPs) or damage‐associated molecular patterns (DAMPs), followed by effector signals (e.g., K⁺/Ca^2^⁺ flux, ATP) to activate caspase‐1‐dependent GSDMD cleavage and subsequent interleukin‐1 beta (IL‐1β)/interleukin‐18 (IL‐18) release.^[^ ^16^ ^]^ Despite GSDMD‐driven macrophage pyroptosis aggravating inflammation and fibrosis in pulmonary,^[^ ^17^ ^]^ hepatic,^[^ ^18^ ^]^ and atherosclerotic^[^ ^19^ ^]^ disease, its role in CKD is not well defined. Previous studies implicate bone marrow‐ or neutrophil‐derived GSDMD in renal fibrosis,^[^ ^20^ ^]^ and TEC‐derived ATP drives macrophage pyroptosis in obstruction models.^[^ ^14^ ^]^ Therefore, the specific function of macrophage GSDMD‐mediated pyroptosis in renal fibrosis requires clarification.

Signal transducer and activator of transcription (STAT) family members regulate the transcription of gasdermin genes during inflammatory responses. STAT1 mediates TEC pyroptosis during acute kidney injury (AKI),^[^ ^21^ ^]^ whereas STAT3 upregulates gasdermin E expression in atherosclerotic macrophages.^[^ ^22^ ^]^ The type III interferons (IFNs), key agonists of the Janus kinase (JAK)/STAT pathway, promote Z‐DNA binding protein 1/caspase‐8/gasdermin C (GSDMC)‐mediated pyroptosis in gut epithelia, impairing mucosal repair and driving inflammatory bowel disease.^[^ ^23^ ^]^ However, the mechanistic interplay between STAT family members and *Gsdmd* transcription in renal macrophages during fibrosis remains uncharacterized, potentially underlying context‐dependent pyroptosis mechanisms.

Using murine models of renal unilateral ischemia‐reperfusion injury (UIRI) and folic acid (FA) nephropathy, we demonstrated that macrophages were the predominant renal GSDMD‐expressing cells, with elevated pyroptosis correlating with fibrosis severity. Macrophage‐specific *Gsdmd* deletion, adoptive transfer of GSDMD‐deficient macrophages, or IFN‐α neutralization ameliorated renal inflammation and fibrosis. Mechanistically, injured TECs‐derived IFN‐α activates the interferon alpha/beta receptor 1 (IFNAR1)/JAK2/STAT2 axis in macrophages. STAT2 formed a complex with interferon regulatory factor 9 (IRF9), directly binding the *Gsdmd* promoter to upregulate its transcription. These findings reveal a TEC‐macrophage IFN‐α/STAT2/IRF9 feedback loop driving pyroptosis and position this axis as a potential therapeutic target for CKD.

## 2 Results

### 2.1 Upregulation of GSDMD and Pyroptosis in Renal Macrophages Correlates with Renal Fibrosis

**Figure 1:**
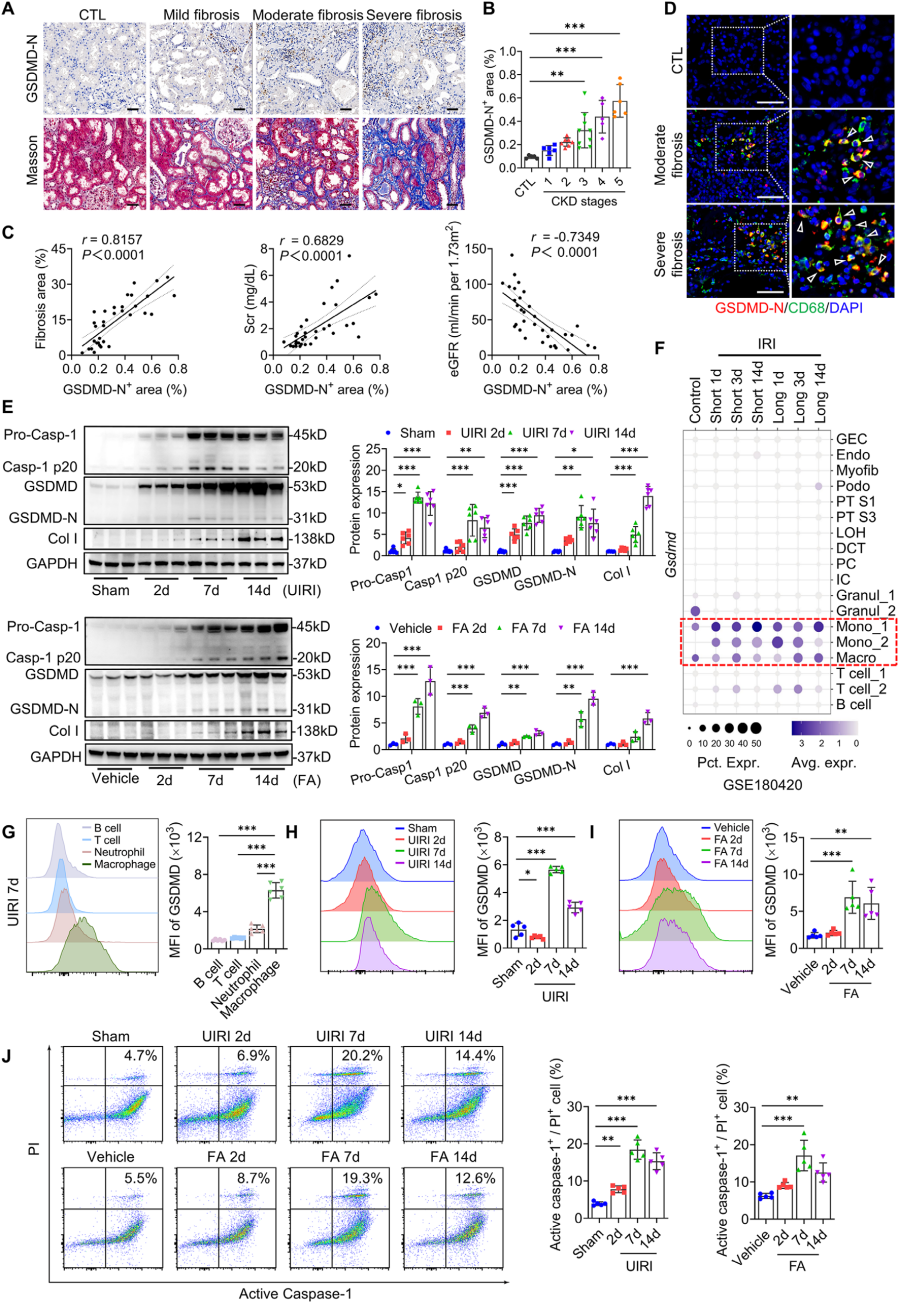
Upregulation of GSDMD and pyroptosis in renal macrophages is associated with renal fibrosis. A) Representative images of GSDMD‐N immunostaining (upper panel) and Masson's staining (lower panel) in human chronic kidney disease (CKD). Bar = 50 µm. B) Quantitative analysis of renal GSDMD‐N expression in different stages of CKD (*n* = 33 for CKD group, *n* = 5 for CTL group). C) The correlation between renal GSDMD‐N and renal fibrosis, serum creatinine (Scr), and estimated glomerular filtration rate (eGFR) in patients with CKD (*n* = 33). D) Representative immunofluorescence images of kidney sections from CKD patients stained for CD68 (green), GSDMD‐N (red), and DAPI (blue; nuclei). Bar = 20 µm. E) Representative immunoblotting and quantitative data of pro‐caspase‐1, cleaved caspase‐1 (p20), GSDMD, GSDMD‐N, and collagen I (Col I) in the kidneys of unilateral ischemia‐reperfusion injury (UIRI) and folic acid (FA) mice (days 2, 7, and 14 after operation or injection, *n* = 3–6) or sham‐operated/vehicle mice (*n* = 3–6). F) The distribution and relative expression of *Gsdmd* in different renal cell types of IRI mice (From sc‐RNA‐seq database GSE180420; *n* = 6 controls, *n* = 6 short, *n* = 6 long IRI samples). GEC, glomerular endothelial cell; Endo, endothelial cell; Myofib, myofibroblast; Podo, podocyte; PT S1, proximal convoluted tubule; PT S3, proximal straight tubule; LOH, loop of Henle; DCT, distal convoluted tubule; PC, principal cell; IC, intercalated cell; Granul, granulocyte; Mono, monocyte; Macro, macrophage; T cell; B cell. G) The mean fluorescence intensity (MFI) and quantitative data of GSDMD expression in B cells, T cells, neutrophils, and macrophages from UIRI‐induced fibrotic kidneys (days 7 after operation, *n* = 5). H,I) The MFI and quantitative data of GSDMD expression in macrophages from UIRI and FA‐induced fibrotic kidneys (days 2, 7, and 14 after operation or injection, *n* = 5) or sham‐operated/vehicle mice (*n* = 5). J) Flow‐cytometric analysis of caspase‐1^+^ propidium iodide (PI)^+^ cells among macrophages from UIRI and FA‐induced fibrotic kidneys (days 2, 7, and 14 after operation or injection, *n* = 5) or sham‐operated/vehicle mice (*n* = 5). ^*^ *p* < 0.05, ^**^ *p* < 0.01, ^***^ *p* < 0.001. Data are presented as mean ± SEM. Statistically significant differences were determined by one‐way ANOVA followed by Dunnett's test. The correlation was analyzed using Pearson correlation.

We first assessed renal GSDMD‐N expression in CKD cohorts (Table S1, Supporting Information). Immunohistochemistry showed pronounced GSDMD‐N elevation in fibrotic kidneys compared to non‐CKD controls, with prominent localization to infiltrating leukocytes (**Figure** **1**A). Quantitative analysis revealed progressive GSDMD‐N upregulation across CKD stages (Figure 1B). Renal GSDMD‐N levels showed significant positive correlations with tubulointerstitial fibrosis severity and serum creatinine, but an inverse association with estimated glomerular filtration rate (eGFR) (Figure 1C), suggesting that aberrant GSDMD activation may represent a pathogenic mechanism in renal fibrogenesis. Further, immunofluorescence revealed predominant co‐localization of GSDMD‐N with CD68⁺ macrophages in fibrotic areas of human CKD kidneys (Figure 1D), indicating macrophages as the major site of GSDMD activation.

To validate these clinical findings, we analyzed GSDMD and its N‐terminal fragment (GSDMD‐N) expression in murine CKD models. Both UIRI and FA‐induced fibrosis kidneys exhibited significant upregulation of *Gsdmd* mRNA (Figure S1, Supporting Information) and increased protein expression and cleavage of both caspase‐1 and GSDMD (Figure 1E), suggesting activation of the pyroptotic pathway. Single‐cell RNA sequencing (sc‐RNA‐seq) of IRI kidneys (GSE180420) identified myeloid‐lineage cells, particularly infiltrating monocytes and macrophages, as the predominant sources of *Gsdmd* transcription (Figure 1F). Flow cytometric quantification confirmed that renal macrophages exhibited the highest GSDMD levels among immune subsets (Figure 1G; Figure S2, Supporting Information). Both UIRI and FA treatment markedly upregulated GSDMD protein in macrophages, peaking at day 7 (Figure 1H,I), coinciding with maximal pyroptosis rates (Figure 1J). Comparative analysis revealed infiltrating macrophages exhibited higher GSDMD expression (Figure S3A–D, Supporting Information) and pyroptosis rates (Figure S3E,F, Supporting Information) than tissue‐resident macrophages. These data establish a pathological link between the upregulation of GSDMD and pyroptosis in macrophages during renal fibrosis progression.

### 2.2 Macrophage‐Specific *Gsdmd* Deletion Ameliorates UIRI‐ and FA‐Induced Renal Fibrosis

To clarify the role of GSDMD in renal fibrosis, we generated macrophage‐specific *Gsdmd* deletion mice (*Gsdmd^ΔLyz2^*) by crossing *Gsdmd^fl/fl^* mice with *Lyz* *2*‐Cre mice (Figure S4A,B, Supporting Information). Macrophage‐specific *Gsdmd* ablation was confirmed using bone marrow‐derived macrophages (BMDMs) differentiated with macrophage colony‐stimulating factor (m‐CSF) (Figure S4C,D, Supporting Information). Both mRNA (Figure S4E, Supporting Information) and protein (Figure S4F, Supporting Information) levels of GSDMD were remarkably reduced in BMDMs from *G* *s* *d* *m* *d^ΔLyz2^* mice compared to *Gsdmd^fl/fl^* controls. Under physiological conditions, genotypes showed no obvious differences in body weight, hepatic function, renal function, or renal histology (Figure S4G–J, Supporting Information).

**Figure 2:**
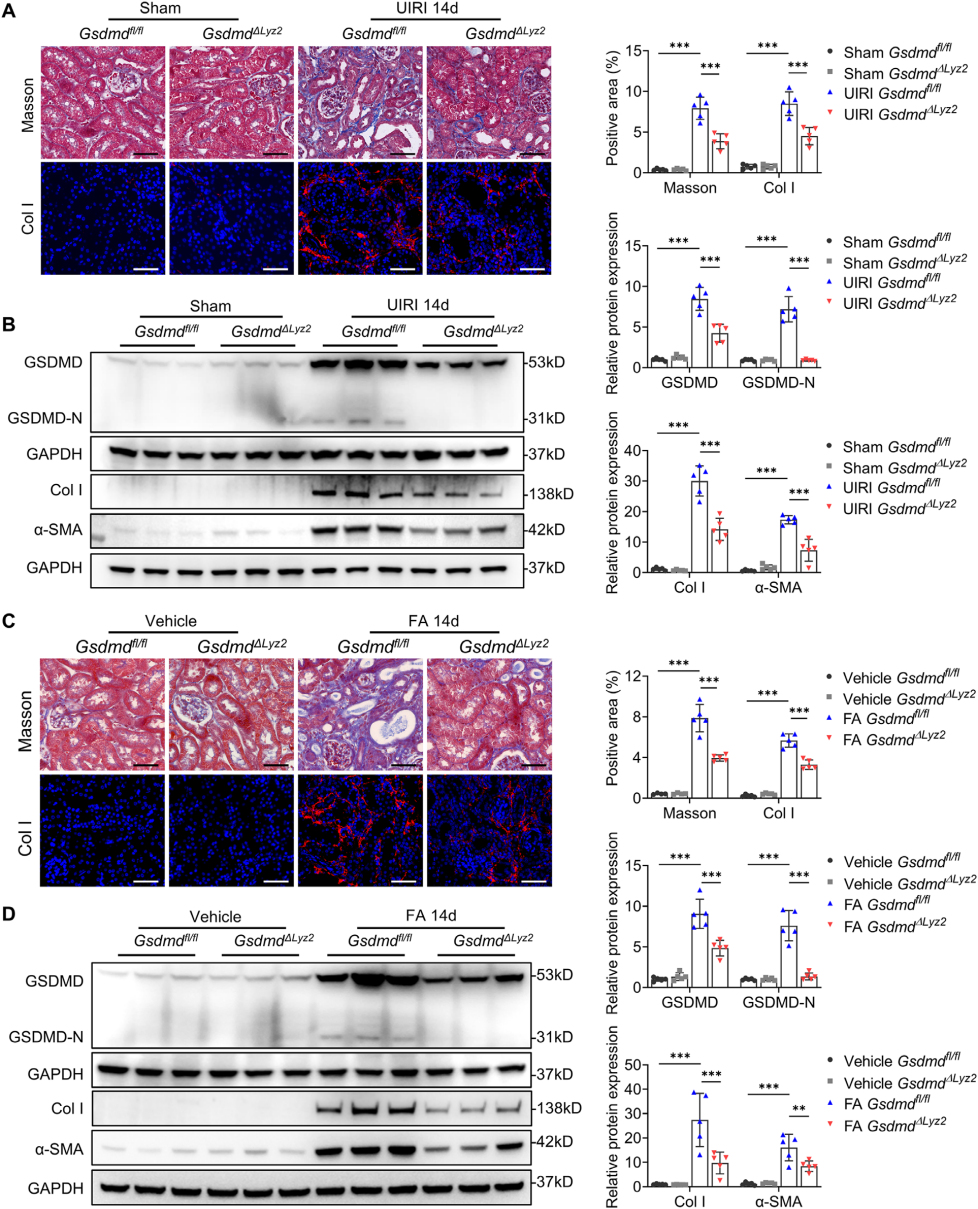
Macrophage‐specific deletion of *Gsdmd* ameliorates the progression of UIRI‐ and FA‐induced renal fibrosis. A) Representative images and quantitative data of Masson's staining and collagen I (Col I) immunostaining in kidney sections at day 14 after UIRI (*n* = 5). Bar = 50 µm. B) Representative immunoblotting and quantitative data of GSDMD, GSDMD‐N, Col I, and alpha‐smooth muscle actin (α‐SMA) protein levels in the kidneys at day 14 after UIRI (*n* = 5). C) Representative images and quantitative data of Masson's staining and Col I immunostaining in kidney sections at day 14 after FA injection (*n* = 4–5). Bar = 50 µm. D) Representative immunoblotting and quantitative data of GSDMD, GSDMD‐N, Col I, and α‐SMA protein levels in the kidneys at day 14 after FA injection (*n* = 5). ^*^ *p* < 0.05, ^**^ *p* < 0.01, ^***^ *p* < 0.001. Data are presented as mean ± SEM. Statistically significant differences were determined by one‐way ANOVA followed by Bonferroni's test.

In the UIRI model, *Gsdmd^ΔLyz2^* mice exhibited decreased renal collagen deposition at day 14 post‐injury compared to *Gsdmd^fl/fl^* littermates (**Figure** **2**A). Macrophage‐specific knockout of *Gsdmd* also suppressed the UIRI‐induced increase in total GSDMD and GSDMD‐N protein levels, and markedly inhibited the upregulation of collagen I (Col I) and alpha‐smooth muscle actin (α‐SMA) (Figure 2B). Similar results were observed in FA models, where *Gsdmd^ΔLyz2^* mice displayed less collagen deposition (Figure 2C) and decreased protein levels of GSDMD, GSDMD‐N, Col I, and a‐SMA compared with *Gsdmd^fl/fl^* mice (Figure 2D).

To exclude the effects of AKI on fibrosis, we subjected *Gsdmd^fl/fl^* and *Gsdmd^ΔLyz2^* mice to bilateral IRI (BIRI). At 24 h post‐injury, both genotypes showed comparable blood urea nitrogen (BUN), serum creatinine, tubular injury scores, and renal kidney injury molecule 1 (KIM‐1) expression (Figure S5A–C, Supporting Information), indicating that macrophage GSDMD deficiency specifically ameliorates CKD progression independent of acute injury mechanisms.

### 2.3 Macrophage‐Specific *Gsdmd* Deletion Attenuates Pyroptosis and Renal Inflammation

**Figure 3:**
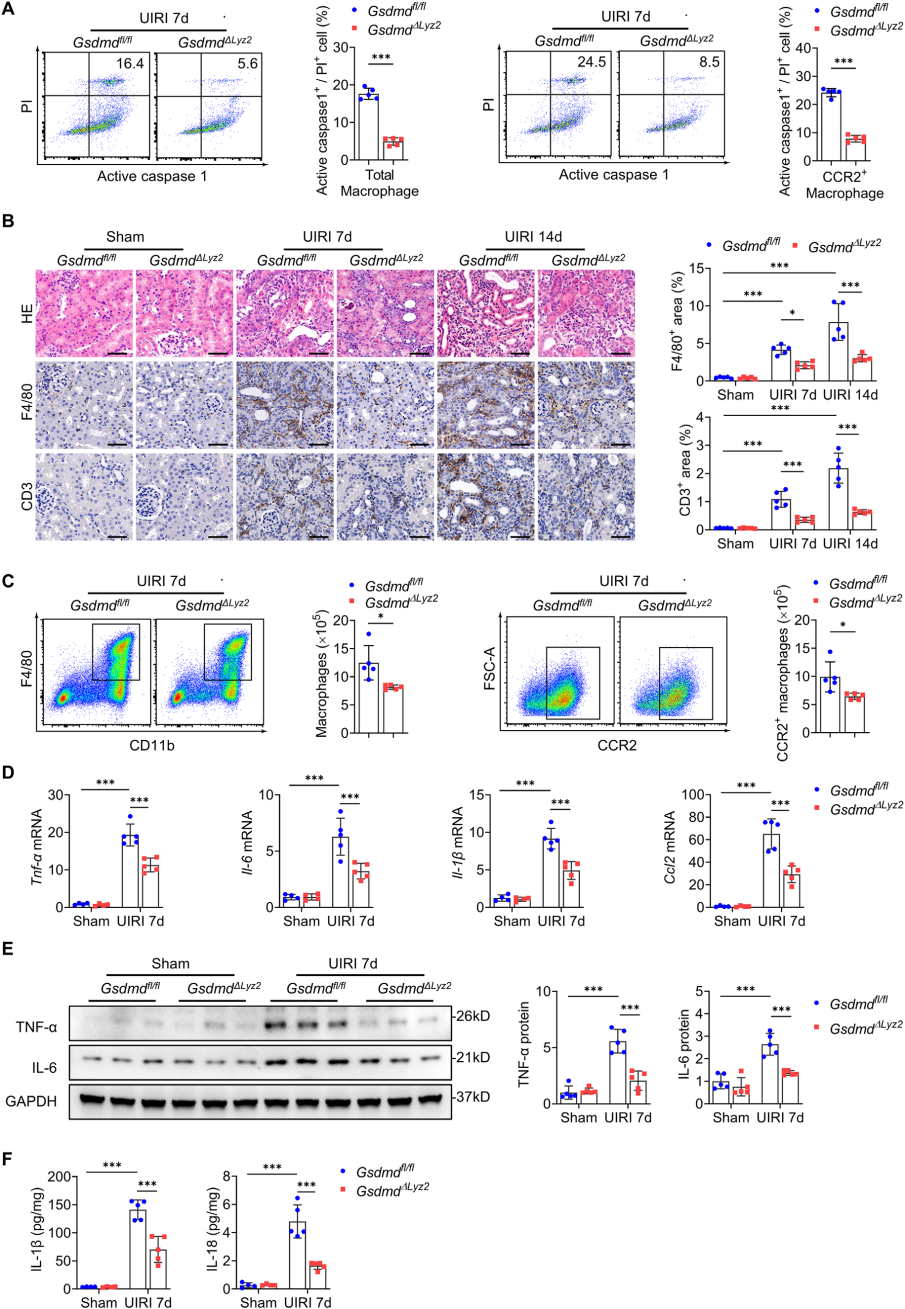
Macrophage‐specific deletion of *Gsdmd* attenuates pyroptosis and renal inflammation. A) Flow‐cytometric analysis of caspase‐1^+^ PI^+^ cells among macrophages and CCR2^+^ macrophages in the kidneys at day 7 after UIRI (*n* = 5). B) Representative images and quantitative data of hematoxylin‐eosin (HE) staining, F4/80, and CD3 immunostaining in kidney sections at days 7 and 14 after UIRI (*n* = 5). Bar = 50 µm. C) Flow‐cytometric analysis of total macrophages and CCR2^+^ macrophages in the kidneys at day 7 after UIRI (*n* = 5). D) Quantitative reverse transcriptase–polymerase chain reaction (qRT‐PCR) of the relative mRNA levels of renal *Tnf‐α*, *Il‐6*, *Il‐1β*, and *Ccl2* at day 7 after UIRI (*n* = 4–5). E) Representative immunoblotting and quantitative data of TNF‐α and IL‐6 protein levels in the kidneys at day 7 after UIRI (*n* = 5). F) ELISA analysis of IL‐1β and IL‐18 levels in the kidney at day 7 after UIRI (*n* = 4–5). ^*^ *p* < 0.05, ^**^ *p* < 0.01, ^***^ *p* < 0.001. Data are presented as mean ± SEM. Statistically significant differences were determined by a 2‐tailed Student's *t*‐test in A and C, and by one‐way ANOVA followed by Bonferroni's test in other panels.

Previous studies have highlighted inflammation as a driver of CKD progression.^[^ ^3^ ^]^ Considering the central role of pyroptosis in the inflammatory cascade,^[^ ^11^ ^]^ we investigated whether macrophage‐specific *Gsdmd* deletion modulates both pathological processes. In the UIRI model, flow cytometry revealed that macrophage‐specific *Gsdmd* deletion significantly suppressed both total macrophage pyroptosis and, in particular, CCR2‐positive (CCR2^+^) infiltrating macrophage pyroptosis, a key fibrosis‐promoting subset,^[^ ^24^ ^]^ compared to *Gsdmd^fl/fl^* controls (**Figure** **3**A; Figure S6A, Supporting Information). Notably, *Gsdmd^ΔLyz2^* mice exhibited a significant reduction in interstitial inflammatory cell infiltration, as evidenced by decreased renal accumulation of F4/80‐positive macrophages and CD3‐positive T lymphocytes (Figure 3B). Flow cytometry quantification further confirmed significant attenuation of total macrophages and CCR2^+^ macrophage populations in the kidneys of *Gsdmd^ΔLyz2^* mice (Figure 3C; Figure S6B, Supporting Information).Consistently, quantitative reverse transcriptase‐polymerase chain reaction (qRT‐PCR) analysis demonstrated reduced mRNA expression of key pro‐inflammatory genes, including *tumor necrosis factor alpha (* *Tnf‐a)*, *interleukin‐6 (* *Il‐6)*, *Il‐1β*, and *C‐C motif ligand 2 (* *Ccl2)*, in *Gsdmd^ΔLyz2^* mice relative to *Gsdmd^fl/f^* controls (Figure 3D), with corresponding decrease in protein levels of TNF‐α, IL‐6, IL‐1β, and IL‐18 in the kidneys (Figure 3E,F). Notably, M1/M2 macrophage polarization ratio remained unaltered following *Gsdmd* ablation (Figure S6C, Supporting Information). Collectively, these findings suggest that GSDMD‐mediated macrophage pyroptosis drives renal inflammation and fibrotic progression through coordinated regulation of inflammatory cell recruitment and cytokine production.

### 2.4 Adoptive Transfer of GSDMD‐Deficient Macrophages Alleviates Renal Inflammation and Fibrosis

To exclude confounding effects of *L* *y* *z* *2*‐Cre activity in other myeloid populations, macrophages in CD45.1 mice were depleted using clodronate liposomes after UIRI surgery and reconstituted with BMDMs from *Gsdmd^fl/fl^* or *Gsdmd^ΔLyz2^* CD45.2 donors (Figure S7A, Supporting Information). Successful engraftment was confirmed by detecting donor‐derived CD45.2^+^ cells in recipient kidneys (Figure S7B, Supporting Information). Notably, transfer of GSDMD‐deficient macrophages significantly reduced renal collagen deposition (Figure S7C, Supporting Information), downregulated fibrotic markers at the mRNA (Figure S7D, Supporting Information) and protein (Figure S7E, Supporting Information) levels, and suppressed renal expression of inflammatory cytokines (Figure S7F,G, Supporting Information). These results demonstrate that macrophage‐expressed GSDMD directly promotes renal inflammation and fibrosis.

### 2.5 Macrophage Pyroptosis is Triggered by Injured TECs via Secreted Mediators

**Figure 4:**
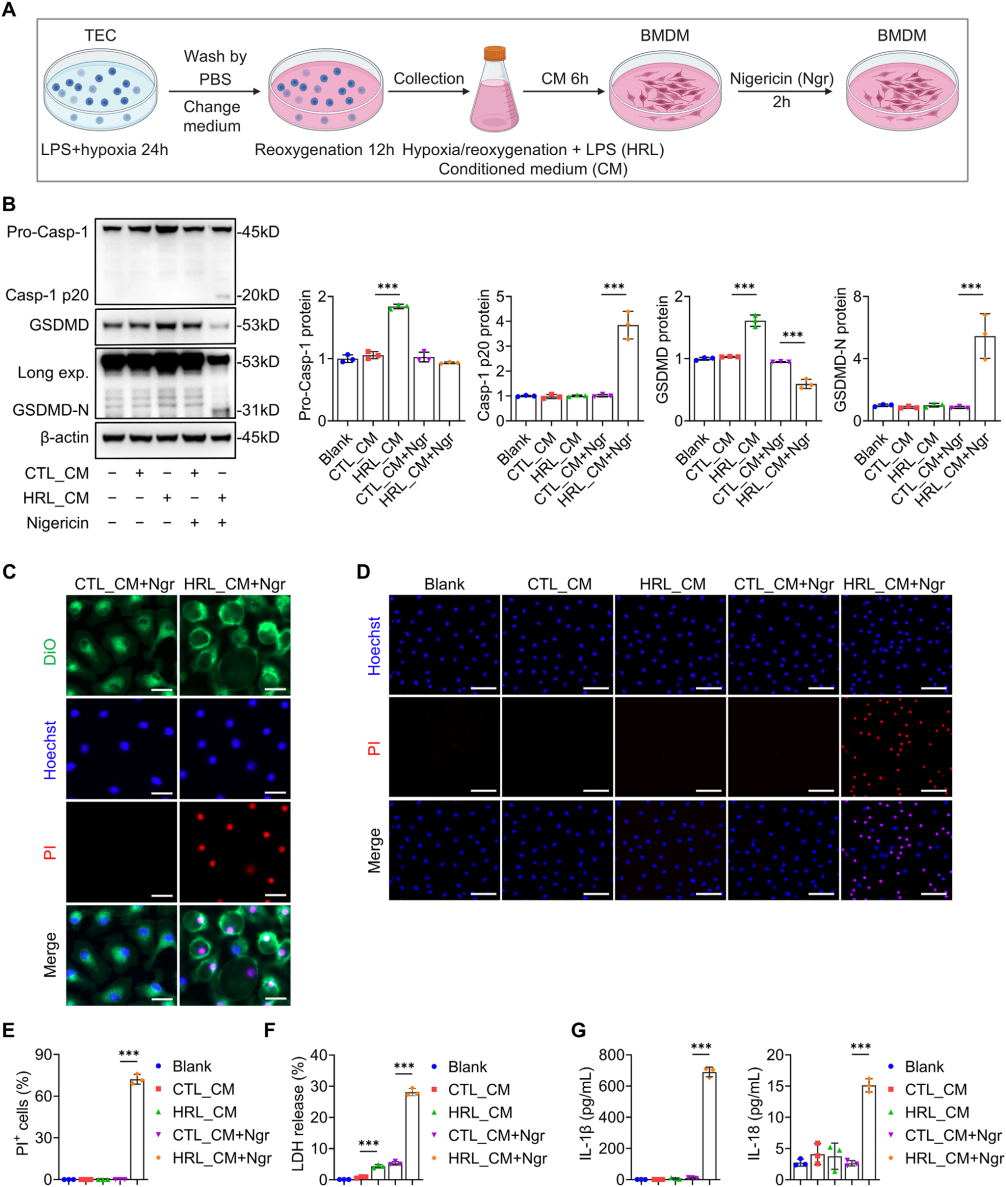
Macrophage pyroptosis is triggered by injured TECs via secreted mediators. A) Experimental design for co‐culture of mouse kidney proximal tubular epithelial cells (TKPTS) and bone marrow‐derived macrophages (BMDMs). TECs were subjected to hypoxia and lipopolysaccharide (LPS) treatment for 24 h, followed by a 12‐h recovery in a fresh medium. Conditioned medium (CM) from injured TECs (HRL CM) and control TECs (CTL CM) was separately collected to treat BMDMs for 6 h, followed by nigericin (10 µm) for 2 h. Figure created with BioRender.com. B) Representative immunoblotting and quantitative data of pro‐caspase‐1, cleaved caspase‐1 (p20), GSDMD, and GSDMD‐N in the BMDMs treated with HRL CM or CTL CM ± nigericin (*n* = 3). C) Immunofluorescence staining of propidium iodide (PI) uptake and plasma membrane integrity in BMDMs treated with HRL CM or CTL CM followed by nigericin. Cells were stained with PI (red) to detect membrane permeabilization and DiO (green) to label the plasma membrane. Nuclei were counterstained with Hoechst (blue). Bar = 20 µm. D) Representative PI staining images of BMDMs treated with HRL CM or CTL CM ± nigericin. Bar = 50 µm. E) Quantification of PI⁺ BMDMs treated with HRL CM or CTL CM ± nigericin (*n* = 3). F) Quantification of LDH release from BMDMs treated with HRL CM or CTL CM ± nigericin (*n* = 3). G) ELISA analysis of IL‐1β and IL‐18 in supernatants of BMDMs treated with HRL CM or CTL CM ± nigericin (*n* = 3). ^*^ *p* < 0.05, ^**^ *p* < 0.01, ^***^ *p* < 0.001. Data are presented as mean ± SEM. Statistically significant differences were determined by one‐way ANOVA followed by Bonferroni's test.

The canonical pyroptotic pathway requires dual activation signals: priming through PAMPs/DAMPs/cytokines, followed by execution signals such as nigericin or ATP.^[^ ^16^, ^25^ ^]^ Considering persistent TEC injury in CKD generating a DAMPs‐rich renal microenvironment, we hypothesized that injured TECs might drive macrophage pyroptosis via secreted mediators. To test this, we established mouse kidney proximal tubular epithelial cells (TKPTS) cultures subjected to hypoxia and lipopolysaccharide (LPS) treatment for 24 h, followed by 12‐h recovery in a fresh medium. Conditioned medium (CM) from injured TECs (HRL CM) and control TECs (CTL CM) was separately collected to treat BMDMs for 6 h, with subsequent nigericin addition to induce potassium efflux (**Figure** **4**A). We found that HRL CM treatment significantly increased the expression of pro‐caspase‐1 and full‐length GSDMD in BMDMs, with nigericin co‐administration further augmenting their cleavage, a hallmark of pyroptosis execution. In contrast, BMDMs exposed to CTL CM showed no elevation in either the full‐length or cleaved proteins (Figure 4B). Morphologically, BMDMs exposed to HRL CM in combination with nigericin exhibited characteristic pyroptotic features, including cellular swelling and plasma membrane bubble‐like protrusions (Figure 4C; Movie S1, Supporting Information), coincide with a significant increase in propidium iodide (PI) positive cells (Figure 4D,E), robust lactate dehydrogenase (LDH) release (Figure 4F), and elevated IL‐1β/IL‐18 secretion (Figure 4G), all indicative of membrane rupture and inflammasome activation. These findings collectively suggest that secretions from injured TECs augment GSDMD expression while promoting GSDMD‐dependent macrophage pyroptosis.

### 2.6 Injured TECs Activate JAK‐STAT Signaling in Macrophages

**Figure 5:**
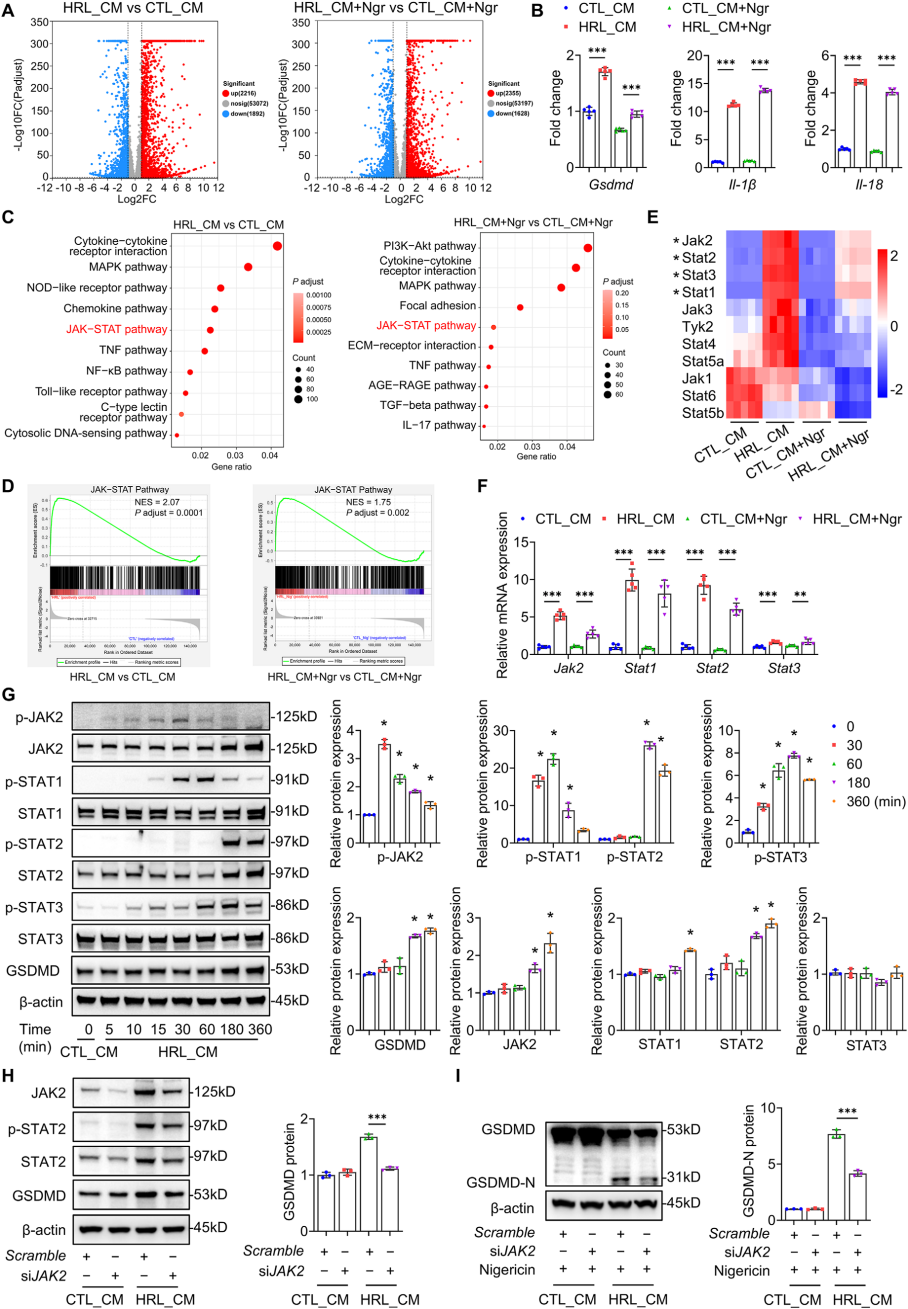
Injured TECs activate JAK‐STAT signaling in macrophages. A) Volcano plots showed the differential expression genes between BMDMs treated with HRL CM and CTL CM (6h) ± nigericin (10 µm, 2h) (*n* = 5). B) RNA‐seq analysis of *Gsdmd*, *Il1β*, and *Il18* expression in BMDMs treated with HRL CM or CTL CM ± nigericin (*n* = 5). C) Kyoto Encyclopedia of Genes and Genomes (KEGG) analysis of the differential signal pathways in BMDMs (HRL CM vs CTL CM, HRL CM + Ngr vs CTL CM + Ngr, *n* = 5). D) Gene Set Enrichment Analysis (GSEA) of the JAK‐STAT pathway in BMDMs (HRL CM vs CTL CM, HRL CM + Ngr vs CTL CM + Ngr, *n* = 5). E) Heatmap of JAK and STAT family genes in BMDMs treated with HRL CM or CTL CM ± nigericin (*n* = 5). F) Quantitative reverse transcriptase–polymerase chain reaction (qRT‐PCR) of the relative mRNA levels of *Jak2*, *Stat1*, *Stat2*, and *Stat3* in BMDMs treated with HRL CM or CTL CM ± nigericin (*n* = 5). G) Representative immunoblotting and quantitative data of the indicated proteins in BMDMs treated with HRL CM or CTL CM (*n* = 3). ^*^ *p* < 0.05 versus CTL_CM. Statistically significant differences were determined by one‐way ANOVA followed by Dunnett's test. H) Representative immunoblotting and quantitative data of JAK2, p‐STAT2, STAT2, and GSDMD in BMDMs treated with HRL CM or CTL CM, with or without *Jak2* knockdown (*n* = 3). I) Representative immunoblotting and quantitative data of GSDMD‐N in BMDMs treated with HRL CM or CTL CM followed by nigericin, with or without *Jak2* knockdown (*n* = 3). ^*^ *p* < 0.05, ^**^ *p* < 0.01, ^***^ *p* < 0.001. Data are presented as mean ± SEM. Statistically significant differences were determined by one‐way ANOVA followed by Bonferroni's test.

To delineate the molecular mechanism underlying injured TECs‐driven macrophage pyroptosis, we performed RNA sequencing (RNA‐seq) on BMDMs treated with HRL CM or CTL CM, with both groups co‐stimulated by nigericin as described above. Volcano plots analysis revealed marked differential expression of genes between groups (**Figure** **5**A). Transcriptomics revealed significant upregulation of pyroptosis‐related genes *Gsdmd*, *Il‐1β*, and *Il‐18* in HRL CM‐treated BMDMs (Figure 5B). Kyoto Encyclopedia of Genes and Genomes (KEGG) pathway enrichment analysis identified significant enrichment of the JAK‐STAT signaling pathway in HRL CM‐treated BMDMs regardless of nigericin stimulation (Figure 5C). This finding was further validated by Gene Set Enrichment Analysis (GSEA), showing pronounced JAK‐STAT enrichment (Figure 5D). Recent studies have implicated the JAK‐STAT pathway in regulating cell pyroptosis,^[^ ^21^, ^26^ ^]^ yet its functional mediation between injured TECs and macrophage pyroptosis remains to be explored. Notably, canonical interferon‐stimulated genes (ISGs) within the JAK‐STAT axis exhibited significant upregulation (Figure S8, Supporting Information), suggesting a possible cytokine‐driven mechanism. Among JAK‐STAT family members, the mRNA levels of *Jak2*, *Stat1*, *Stat2*, and *Stat3* were significantly increased in HRL CM‐treated BMDMs, irrespective of nigericin exposure (Figure 5E). qRT‐PCR confirmed significant elevation of these core components in HRL CM‐treated BMDMs compared to CTL CM counterparts (Figure 5F). Western blot analysis further showed time‐dependent upregulation and phosphorylation of JAK2/p‐JAK2, STAT1/p‐STAT1, STAT2/p‐STAT2, and p‐STAT3 in HRL CM‐treated BMDMs, paralleling GSDMD expression (Figure 5G). Moreover, *Gsdmd* expression in primary kidney macrophages from IRI mice (GSE75808 dataset) exhibited strong positive correlations with *Stat1* (Pearson's r = 0.6881, *P* = 0.0032) and *Stat2* (r = 0.6266, *P* = 0.0094), but not *Stat3* (r = 0.4072, *P* = 0.1175) (Figure S9, Supporting Information). Importantly, *Jak2* knockdown via siRNA in BMDMs attenuated HRL CM‐induced upregulation of STAT2/p‐STAT2 and GSDMD/GSDMD‐N protein levels (Figure 5H,I), confirming JAK‐STAT signaling as a critical regulator of macrophage pyroptosis.

### 2.7 STAT2‐IRF9 Complex Activates *Gsdmd* Transcription

**Figure 6:**
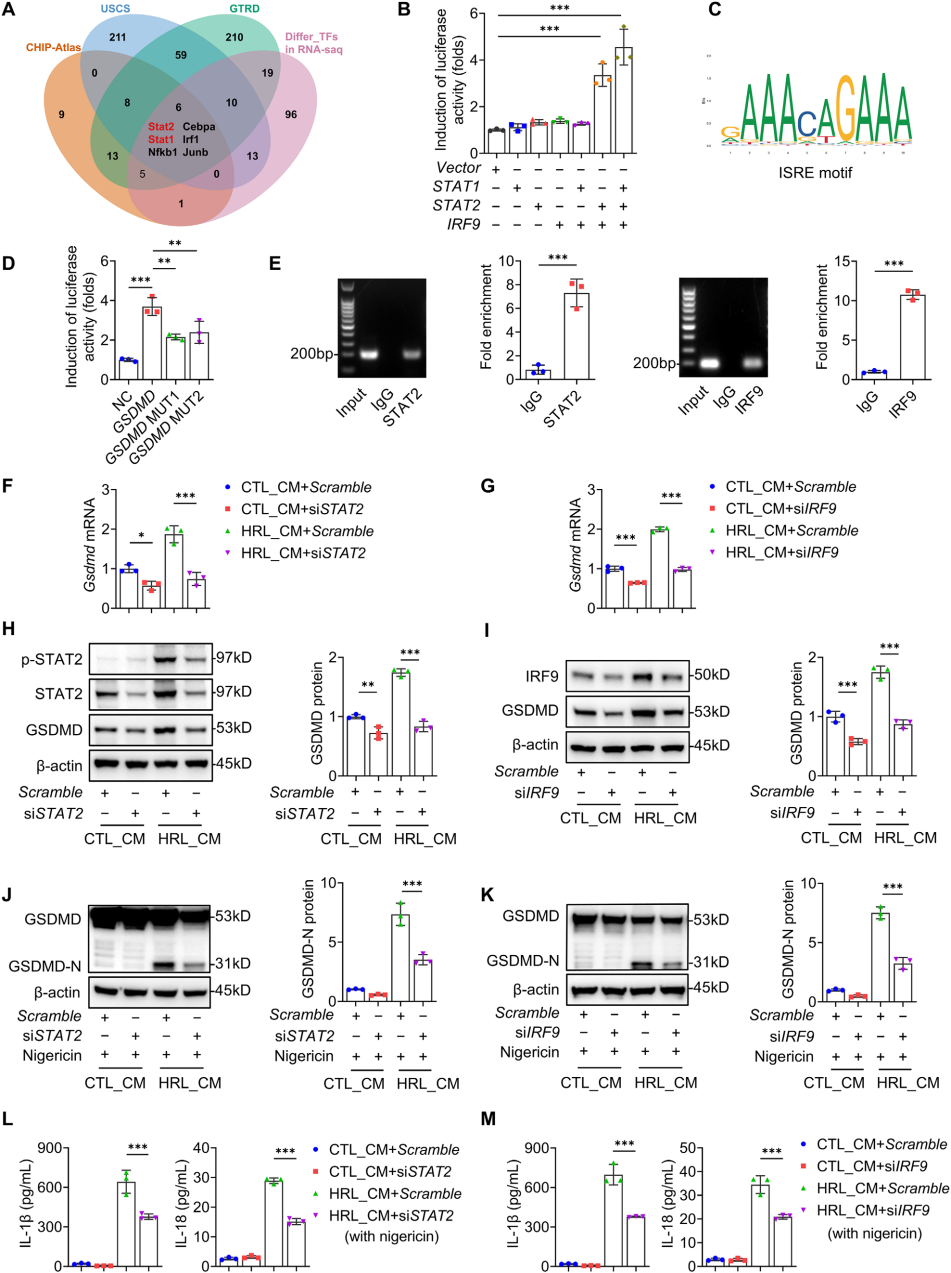
STAT2‐IRF9 complex targets the *Gsdmd* promoter and activates transcription. A) The transcription factors binding to *Gsdmd* promoter regions were predicted by the bioinformatics analysis (GTRD, USCS, and CHIP‐Atlas databases), and then integrated with RNA‐seq‐derived differential transcription factors. B) The luciferase reporter gene assay on 293 T cells transfected with a mouse *GSDMD* promoter‐driven luciferase vector (0.5 µg) and pCMV‐*STAT1*, the pCMV‐*STAT2*, the pCMV‐*IRF9*, or the pCMV‐NC (0.5 µg). After 48 h, the luciferase activity was measured and normalized to Renilla activity (*n* = 3). C) The sequence logo of mouse *Stat2* transcription factor binding sites. D) The luciferase reporter gene assay on 293 T cells transfected with a mouse *GSDMD* or mutant *GSDMD* promoter‐driven luciferase vector (0.5 µg) and the pCMV‐*STAT2* plus with the pCMV‐*IRF9* or pCMV‐NC (0.5 µg). After 48 h, the luciferase activity was measured and normalized to Renilla activity (*n* = 3). E) ChIP analysis of STAT2 and IRF9 binding at the *Gsdmd* promoter. IgG served as a negative control (*n* = 3). F,G) Quantitative reverse transcriptase–polymerase chain reaction (qRT‐PCR) of the relative mRNA levels of *Gsdmd* in BMDMs treated with HRL CM or CTL CM (6h), with or without *Stat2/Irf9* knockdown (*n* = 3). H–K) Representative immunoblotting and quantitative data of indicated proteins in BMDMs treated with HRL CM or CTL CM (6h) ± nigericin (10 µm, 2h), with or without *Stat2/Irf9* knockdown (*n* = 3). L,M) ELISA analysis of IL‐1β and IL‐18 in the supernatants of BMDMs treated with HRL CM or CTL CM followed by nigericin, with or without *Stat2/Irf9* knockdown (*n* = 3). ^*^ *p* < 0.05, ^**^ *p* < 0.01, ^***^ *p* < 0.001. Data are presented as mean ± SEM. Statistically significant differences were determined by one‐way ANOVA followed by Bonferroni's test.

To elucidate the transcriptional regulation mechanism of *Gsdmd* by JAK‐STAT signaling, we performed integrated bioinformatics analysis with experimental validation. By cross‐referencing transcription factor prediction databases (GTRD, UCSC, and CHIP‐Atlas) with RNA‐seq‐derived differential transcription factors, we identified six potential regulators of *Gsdmd* transcription, with STAT1 and STAT2 as top candidates (**Figure** **6**A). STAT2 requires IRF9 for DNA binding,^[^ ^27^ ^]^ forming the STAT2‐IRF9 complex that maintains basal expression of ISGs.^[^ ^28^ ^]^ This complex associates with STAT1 to constitute interferon‐stimulated gene factor 3 (ISGF3) upon Type I/III IFN stimulation.^[^ ^29^ ^]^ Functional validation using dual‐luciferase reporter assays in 293T cells revealed synergistic transcriptional activation: co‐transfection of STAT1/STAT2/IRF9 increased luciferase activity by 4‐fold compared to controls, while individual factors showed no significant effect (Figure 6B). Intriguingly, STAT2/IRF9 co‐transfection achieved 70% of maximal activity (Figure 6B), suggesting their dominant role in *Gsdmd* transcription. JASPAR analysis revealed conserved STAT2‐binding motifs in the *Gsdmd* promoter with relative scores exceeding 85% (Figure 6C, Table S2, Supporting Information), which were abolished by mutation of the conserved interferon‐stimulated response element (ISRE) motifs (Figure 6D). Furthermore, chromatin immunoprecipitation quantitative PCR (ChIP‐qPCR) in macrophages revealed significant enrichment of both STAT2 and IRF9 at the *Gsdmd* promoter, directly validating their transcriptional regulatory role (Figure 6E).

To confirm the transcriptional function of the STAT2‐IRF9 complex in GSDMD regulation, we performed siRNA‐mediated knockdown of *Stat2* or *Irf9* in BMDMs. This reduced *Gsdmd* mRNA abundance (Figure 6F,G) and GSDMD protein levels in HRL CM‐treated BMDMs (Figure 6H,I), suppressed GSDMD cleavage (Figure 6J,K), and inhibited IL‐1β/IL‐18 secretion (Figure 6L,M). These findings establish STAT2–IRF9 complex as the master transcriptional regulator of *Gsdmd* in macrophages.

### 2.8 Injured TECs‐Derived IFN‐α Induces Macrophage Pyroptosis via IFNAR1 Signaling

**Figure 7:**
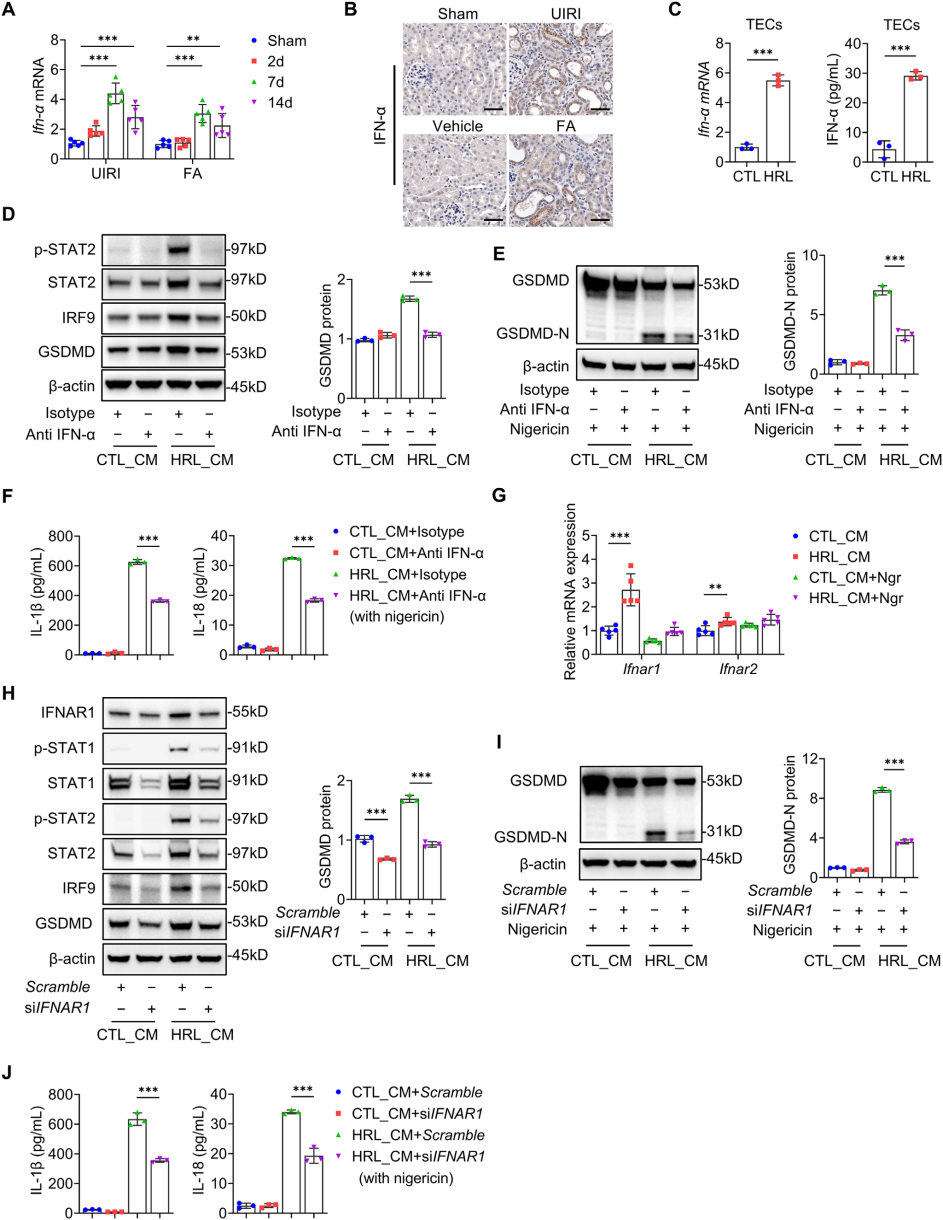
Injured‐TECs‐derived IFN‐α induces macrophage pyroptosis via IFNAR1 signaling. A) Quantitative reverse transcriptase‐polymerase chain reaction (qRT‐PCR) of the relative mRNA levels of *Ifn‐α* in the kidneys of UIRI and FA mice (*n* = 5). B) Immunohistochemistry staining of IFN‐α in kidney sections from UIRI and FA mice. Bar = 50 µm. C) qRT‐PC of the relative mRNA levels of *Ifn‐α* and ELISA analysis of IFN‐α in the supernatant of TECs treated with hypoxia/reoxygenation (H/R) + LPS (HRL) or CTL (*n* = 3). D) Immunoblotting and quantification of indicated proteins in BMDMs treated with HRL CM or CTL CM (6h), with or without anti‐IFN‐α (*n* = 3). E) Immunoblotting and quantification of GSDMD‐N in BMDMs treated with HRL CM or CTL CM (6h) followed by nigericin (10 µm, 2h), with or without anti‐IFN‐α (*n* = 3). F) ELISA analysis of IL‐1β and IL‐18 in supernatants from BMDMs treated as in (E) (*n* = 3). G) qRT‐PCR of the relative mRNA levels of *Ifnar1* and *Ifnar2* in BMDMs treated with HRL CM or CTL CM ± nigericin (*n* = 5). H) Representative immunoblotting and quantitative data of the indicated proteins in BMDMs treated with HRL CM or CTL CM, with or without *Ifnar1* knockdown (*n* = 3). I) Representative immunoblotting and quantitative data of GSDMD‐N in BMDMs treated with HRL CM or CTL CM followed by nigericin, with or without *Ifnar1* knockdown (*n* = 3). J) ELISA analysis of IL‐1β and IL‐18 in the supernatants of BMDMs treated as in (I) (*n* = 3). ^*^ *p* < 0.05, ^**^ *p* < 0.01, ^***^ *p* < 0.001. Data are presented as mean ± SEM. Statistically significant differences were determined by one‐way ANOVA followed by Bonferroni's test.

Both Type I (IFN‐α/β) and III IFNs (IFN‐λ2/3) are recognized activators of STAT2 signaling.^[^ ^30^ ^]^ Although renal IFN‐α elevation has been reported in IRI‐ and cisplatin‐induced AKI models,^[^ ^31^, ^32^ ^]^ the role of IFN subtypes in fibrotic progression remains elusive. In our study, *Ifn‐a* transcripts (**Figure** **7**A) and protein levels were significantly upregulated in fibrotic kidneys post‐UIRI and FA injury, with predominant localization to injured tubular epithelial cells and minimal interstitial staining (Figure 7B). This upregulation was mirrored by elevated *Ifn‐a* mRNA and secreted protein in TECs subjected to H/R‐ and LPS‐challenge (Figure 7C). By contrast, *Ifn‐β*, *Ifn‐λ2*, and *Ifn‐λ3* mRNA expression remained unaltered both in vivo and in vitro (Figure S10A,B, Supporting Information), pinpointing IFN‐α as the predominant interferon subtype in this pathological context.

Anti‐IFN‐α antibody neutralization of TECs‐derived HRL CM markedly suppressed STAT2 phosphorylation and expression of STAT2, IRF9, and GSDMD in BMDMs (Figure 7D), reduced GSDMD cleavage (Figure 7E), and decreased IL‐1β/IL‐18 release (Figure 7F). These data demonstrate that tubular epithelial‐derived IFN‐α promotes macrophage pyroptosis via the STAT2/IRF9 axis. Given that IFN‐α secreted by injured TECs may regulate macrophage responses via binding to IFN‐α/β receptor (IFNAR) complex (IFNAR1 and IFNAR2), we investigated this possibility. Intriguingly, CM from injured TECs remarkably enhanced *Ifnar1* mRNA (Figure 7G) and IFNAR1 protein expression in BMDMs (Figure 7H), while *Ifnar2* mRNA upregulation was minimal (Figure 7G). Moreover, *Gsdmd* expression in primary kidney macrophages from IRI mice (GSE75808 dataset) exhibited a strong positive correlation with *Ifnar1* (Pearson's r = 0.7227, *P =* 0.0016) (Figure S10C, Supporting Information). *Ifnar1* knockdown markedly blocked STAT1/STAT2 phosphorylation and reduced STAT1, STAT2, IRF9, and GSDMD protein levels in BMDMs exposed to HRL CM (Figure 7H). Furthermore, *Ifnar1* silencing suppressed GSDMD cleavage (Figure 7I) and IL‐1β/IL‐18 secretion (Figure 7J), hallmarks of pyroptosis. Our findings collectively suggest that injured TEC‐derived IFN‐α orchestrates macrophage pyroptosis through an IFNAR1‐dependent mechanism, revealing a novel epithelial‐immune interaction axis in renal fibrogenesis.

### 2.9 IFN‐α Neutralizing Antibody Attenuates Macrophage Pyroptosis, Renal Inflammation, and Fibrosis Induced by UIRI

**Figure 8:**
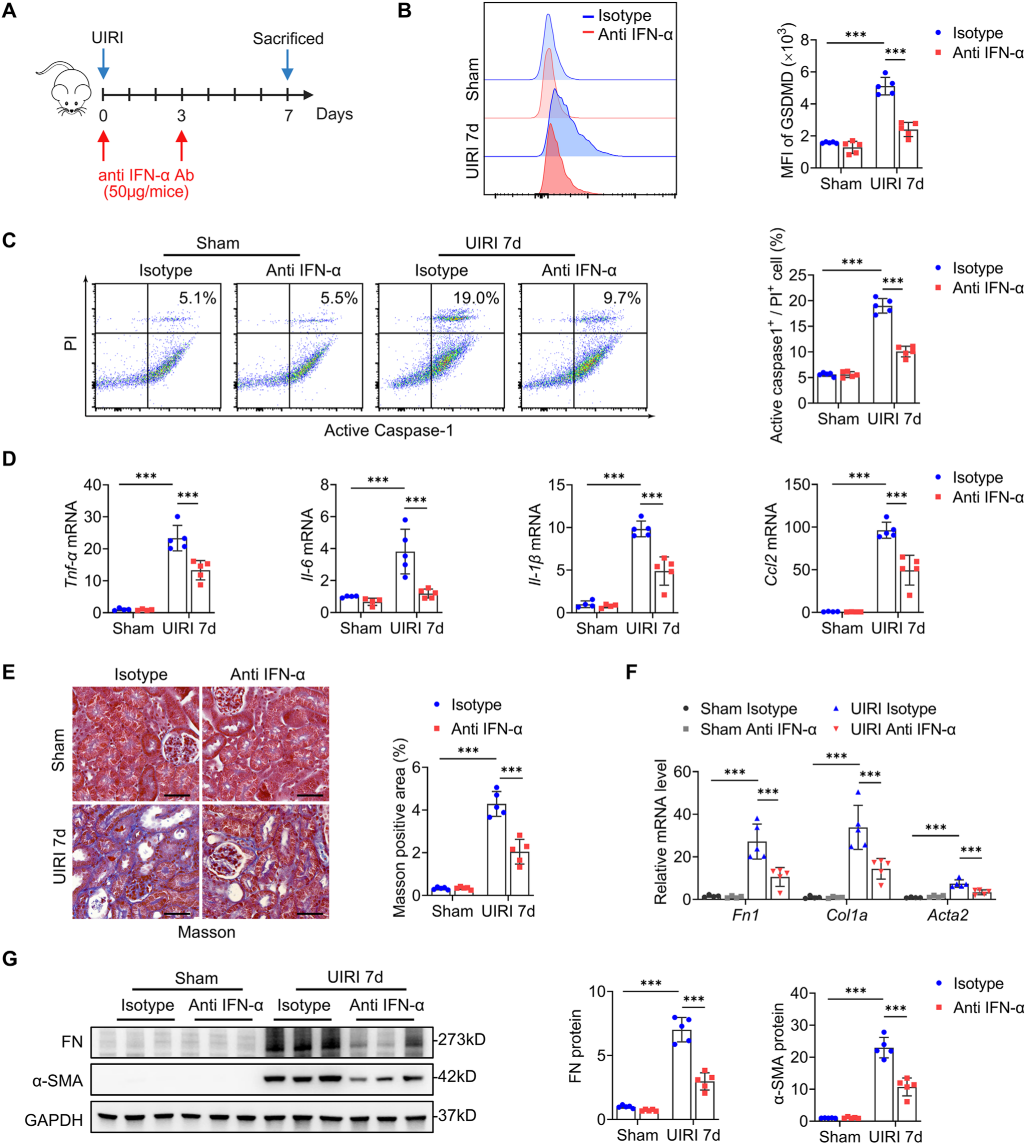
IFN‐α neutralization attenuates renal UIRI‐induced macrophage pyroptosis, inflammation, and fibrosis. A) Schematic of the experimental design; the red arrow indicates the time of injecting IFN‐α antibody or isotype IgG. B) The MFI and quantitative data of GSDMD expression among macrophages in the kidneys at day 7 after UIRI (*n* = 5). C) Flow‐cytometric analysis of caspase‐1^+^ PI^+^ cells among macrophages in the kidneys at day 7 after UIRI (*n* = 5). D) qRT‐PCR of the relative mRNA levels of renal *Tnf‐α*, *Il‐6*, *Il‐1β*, and *Ccl2* at day 7 after UIRI (*n* = 4–5). E) Representative images and quantitative data of Masson's staining in kidney sections at day 7 after UIRI (*n* = 5). Bar = 50 µm. F) qRT‐PCR of the relative mRNA levels of renal *Fn1*, *Col1a*, and *Acta2* at day 7 after UIRI (*n* = 4–5). G) Representative immunoblotting and quantitative data of fibronectin (FN) and α‐SMA protein levels in the kidneys at day 7 after UIRI (*n* = 5). ^*^ *p* < 0.05, ^**^ *p* < 0.01, ^***^ *p* < 0.001. Data are presented as mean ± SEM. Statistically significant differences were determined by one‐way ANOVA followed by Bonferroni's test.

To validate the paracrine role of IFN‐α in macrophage pyroptosis and renal fibrosis, we intravenously administered IFN‐α neutralizing antibody or isotype control into 8‐week‐old male wild‐type C57BL/6J mice 30 min prior to UIRI challenge, followed by a second dose 3 days after surgery (**Figure** **8**A). As expected, IFN‐α neutralization markedly attenuated UIRI‐induced macrophage GSDMD expression and pyroptosis (Figure 8B,C). Consistently, qRT‐PCR revealed a significant reduction in renal inflammatory cytokines (*Tnf‐a*, *Il‐6*, and *Il‐1β*) and the chemokine *Ccl2* mRNA levels in the neutralizing antibody group relative to the isotype control (Figure 8D). Furthermore, neutralizing antibody treatment remarkably reduced renal interstitial collagen deposition and the expression of fibrotic markers like fibronectin and α‐SMA (Figure 8E–G). Collectively, these results demonstrated that IFN‐α released from injured TECs drives renal inflammation and fibrosis through a paracrine mechanism involving macrophage pyroptosis induction.

## 3 Discussion

In this study, we demonstrated elevated GSDMD predominantly expressed in renal macrophages of CKD patients and murine models, with its active fragment GSDMD‐N showing a positive correlation with fibrosis severity. Macrophage‐specific *Gsdmd* ablation, adoptive transfer of GSDMD‐deficient macrophages, or IFN‐α neutralization attenuated renal inflammation and fibrogenesis. Mechanistically, IFN‐α secreted by injured TECs activated the IFNAR1/JAK2/STAT2 axis in macrophages, promoting STAT2‐IRF9 complex formation that directly bound the *Gsdmd* promoter to boost its transcription. Collectively, injured TEC‐derived IFN‐α established a paracrine TEC‐macrophage feedback loop via GSDMD‐mediated macrophage pyroptosis, exacerbating renal inflammation and fibrosis, thus uncovering a novel CKD pathogenesis mechanism.

GSDMD contributes to various chronic pathologies, including autoimmune disease, atherosclerosis, pulmonary and hepatic fibrosis, antitumor immunity,^[^ ^33^ ^]^ and its global knockout can attenuate renal fibrosis,^[^ ^20^, ^34^ ^]^ although its cell‐type‐specific roles in CKD remain elusive. Contrary to reports showing GSDMD‐mediated macrophage pyroptosis promotes muscle repair,^[^ ^35^ ^]^ our findings align with skin, pulmonary, and hepatic fibrosis attenuation by inhibiting GSDMD‐mediated macrophage pyroptosis in mouse models.^[^ ^17^, ^18^, ^36^ ^]^ Notably, Wang et al. employed chimera experiments to reveal bone marrow‐derived cell pyroptosis drives renal fibrosis, whereas further studies reported neutrophil‐, but not macrophage‐, specific *Gsdmd* deficiency reduces renal fibrosis.^[^ ^20^ ^]^ Our study revealed that activated GSDMD‐N predominantly localized to macrophages in human CKD specimens.  Moreover, single‐cell transcriptomics and flow cytometry confirmed renal macrophages as the primary *Gsdmd*‐expressing leukocyte subset, with peak pyroptotic activity at 7 days post‐injury. Importantly, macrophage‐specific *Gsdmd* ablation reduces renal fibrosis independently of IRI‐induced acute tubular injury, coincident with findings in pulmonary and hepatic fibrosis,^[^ ^17^, ^18^ ^]^ thereby positioning macrophage pyroptosis as a conserved fibrotic mechanism across organs.

GSDMD represents the central mediator of gasdermin family‐driven inflammation in cytokine release syndrome, sepsis, and acute organ injury.^[^ ^11^ ^]^ While IL‐1β and IL‐18 release depend on GSDMD pore formation,^[^ ^37^ ^]^ our results revealed distinct regulatory mechanisms in renal fibrosis. Specifically, genetic or adoptive ablation of macrophage GSDMD reduced pyroptosis, inflammatory infiltration, and cytokine secretion, without altering macrophage polarization, suggesting its primary role in executing pyroptosis rather than regulating polarization. This contrasts with Gong's observation that the pyroptosis inhibitor dimethyl fumarate suppresses both M1 macrophage polarization and pyroptosis via caspase1/GSDMD signaling pathway in AKI,^[^ ^38^ ^]^ highlighting context‐dependent functional heterogeneity of GSDMD. Moreover, the immunogenicity of pyroptosis is highlighted by macrophage‐derived pyroptotic vesicles transmitting active GSDMD pores to adjacent cells, inducing a domino effect of secondary death and amplifying local inflammation.^[^ ^39^ ^]^

Mechanisms initiating renal macrophage pyroptosis are largely unknown. Prior studies demonstrate that DAMPs (e.g., high mobility group box 1 (HMGB1), interleukin‐36, necrotic DNA) released from injured TECs exemplify classical pyroptosis triggers to activate macrophage inflammasome and amplify TECs‐driven inflammation in CKD.^[^ ^40^, ^41^, ^42^ ^]^ By using the co‐culture experiments, we revealed that injured TECs‐derived conditional medium activates macrophage JAK/STAT pathway and GSDMD‐mediated pyroptosis upon nigericin stimulation. STAT1 is implicated in hypoxia/reoxygenation‐induced tubular pyroptosis,^[^ ^21^ ^]^ while JAK2^VF^ mutations enhance absent in melanoma 2 (AIM2) inflammasome‐driven pyroptosis in macrophages.^[^ ^26^ ^]^ Intriguingly, our dual‐luciferase assays revealed the ISGF3 complex (STAT1/STAT2/IRF9) as the most efficient *Gsdmd* promoter activator. Functional analyses indicated that STAT2/IRF9 subcomplex and ISGF3 drove robust transcription, whereas STAT1 or STAT2 alone displayed minimal activity. ChIP‐qPCR directly confirmed STAT2 and IRF9 binding at the endogenous *Gsdmd* promoter. Mechanistically, IFN signaling activates JAK‐STAT through STAT1/STAT2 phosphorylation, recruiting IRF9 to form ISGF3.^[^ ^29^ ^]^ This complex regulates inflammatory genes through ISRE‐binding elements.^[^ ^29^ ^]^ Consistent with this hierarchy, *Stat1* genetic ablation only mildly reduced GSDMD mRNA and protein levels (*data not shown*). In contrast, *Stat2* or *Irf9* deficiency suppressed both transcripts and proteins under control conditioned medium, indicating their essential role in basal *Gsdmd* transcription during homeostasis and functional compensation by the STAT2/IRF9 subcomplex. This is consistent with recent findings of STAT2/IRF9 complex‐mediated transcriptional activation independent of STAT1,^[^ ^28^, ^43^, ^44^, ^45^ ^]^ suggesting functional compensation in homeostatic conditions.

The JAK‐STAT2 axis, canonically activated by Type I (IFN‐α/β) and III (IFN‐λ2/3) IFNs, contributes to viral infection and autoinflammatory disorders.^[^ ^30^ ^]^ Renal IFN‐α levels were elevated in AKI models of IRI and cisplatin nephrotoxicity,^[^ ^31^, ^32^ ^]^ with IFN‐α neutralization alleviating inflammatory infiltration and renal damage.^[^ ^32^ ^]^ Notably, type III IFNs induce intestinal epithelial GSDMC‐mediated pyroptosis.^[^ ^23^ ^]^ However, whether interferons regulate renal macrophage pyroptosis remains undefined. Our data demonstrated that IFN‐α, but not IFN‐β/λ2/λ3, was the predominant interferon subtype in fibrotic kidneys, primarily localized within injured TECs, peaking concurrently with macrophage pyroptotic activity. Hypoxia/reoxygenation and LPS‐challenged TECs recapitulated this pattern, with *Ifn‐*β, *Ifn‐λ2*, and *Ifn‐λ3* expression remaining unaltered. Mechanistically, IFN‐α signaled via upregulated IFNAR1 in macrophages; either IFN‐α neutralization or *Ifnar1* knockdown reduced both GSDMD expression and its cleavage, establishing IFN‐α/IFNAR1 as a critical regulator of this pyroptotic cascade. Since injured TECs release various DAMPs,^[^ ^40^, ^41^, ^42^ ^]^ IFN‐α likely acts collaboratively with other TEC‐derived factors to amplify pyroptosis. Consistent with this notion, in vivo neutralization of IFN‐α partially attenuated UIRI‐induced macrophage pyroptosis and fibrosis. Moreover, IFNAR1 inhibition by antibody has been shown to mitigate AKI‐driven renal fibrosis.^[^ ^46^ ^]^ Together, these findings highlight the IFN‐α/IFNAR1 axis as a key mediator of TEC‐macrophage crosstalk in fibrosis.

Limited studies link Type I IFNs to pyroptosis. sc‐RNA‐seq revealed interferon‐inducible ISG macrophages with upregulated GSDMD in abdominal aortic aneurysms,^[^ ^47^ ^]^ while IFN‐α combined with Src homology region 2 domain‐containing phosphatase‐2 (SHP2) inhibitor SHP099 caused pyroptosis in renal cell carcinoma through nuclear factor kappa‐light‐chain‐enhancer of activated B cells (NF‐κB) pathway activation.^[^ ^48^ ^]^ Importantly, targeting type I IFNs has emerged as a promising and effective therapeutic strategy, with a particular emphasis on IFNAR1.^[^ ^49^, ^50^ ^]^ Among these strategies, anifrolumab, an IFNAR1 antagonist, is FDA‐approved for lupus. However, type I IFNs induce non‐canonical IL‐1β secretion in lupus monocytes independently of GSDMD‐mediated pyroptosis,^[^ ^51^ ^]^ suggesting that IFN‐α regulates macrophages through mechanisms beyond pyroptosis, warranting further study in renal fibrosis. Our present findings delineate a paracrine TEC‐macrophage axis wherein IFN‐α derived from injured TECs induces macrophage pyroptosis via JAK2/STAT2‐IRF9‐GSDMD signaling, highlighting the therapeutic potential of targeting IFN‐α/IFNAR1 axis in CKD.

While we demonstrated a role for TEC‐derived IFN‐α in macrophage pyroptosis, the spatial dynamics of IFN‐α action (local tubulointerstitial vs systemic) require clarification. Potential crosstalk between pyroptotic macrophages and other cell types (e.g., endothelial cells, fibroblasts) in the fibrotic microenvironment, such as through IL‐1β or HMGB1 secretion, warrants further exploration.

## 4 Conclusion

In summary, our study demonstrates that macrophage GSDMD‐mediated pyroptosis critically drives fibrosis progression through a TEC‐macrophage IFN‐α/IFNAR1/JAK2/STAT2/IRF9 axis. Targeting this pathway represents a novel therapeutic strategy against renal fibrosis progression.

## 5 Experimental Section

### Human Kidney Specimens

Human kidney specimens were collected from thirty‐three CKD patients with primary glomerulonephritis. All cases were diagnosed at the First Affiliated Hospital of Sun Yat‐sen University from January 2022 to December 2024, with written informed consent. Demographic and clinical data at the initial biopsy are shown in Table S1 (Supporting Information). The estimated glomerular filtration rate (eGFR) was calculated using the CKD‐EPI equation. Renal fibrosis severity was determined by the Masson trichrome staining and semi‐quantitation (no fibrosis; mild: < 25%; moderate‐mild: 25%–50%; severe: > 50%) according to a previous report.^[^ ^52^ ^]^ Non‐tumor renal tissues from five patients with renal cell carcinoma were used as controls. This study was conducted in accordance with the principles of the Declaration of Helsinki and was approved by the First Affiliated Hospital of Sun Yat‐Sen University Institutional Review Board (project number: [2016]215‐1).

### Animals

Male wild‐type C57BL/6 mice and *Gsdmd^fl/fl^* mice were purchased from GemPharmatech (Foshan, Guangdong, China). Macrophage‐specific *Gsdmd* deletion mice (*Gsdmd^ΔLyz2^*) and littermate controls (*Gsdmd^fl/f^*) on a C57BL/6 background were generated by crossing with *L* *y* *z* *2*‐cre mice (Jackson Laboratory, stock#T003822). All mice (male, 8–12 weeks) were housed in specific pathogen‐free facilities at the Laboratory Animal Center of Sun Yat‐Sen University. The animal study protocols were approved by the Institutional Animal Care and Use Committee of Yat‐Sen University (project numbers: 2023000489, 2023000877).

For the UIRI model, mice were anesthetized and subjected to unilateral renal pedicle clamping for 35 min at 37 °C, with sham‐operated mice as controls.^[^ ^24^, ^53^ ^]^ For the BIRI model, mice were anesthetized, and bilateral renal pedicles were clamped for 30 min at 37 °C.^[^ ^54^ ^]^ For the FA model, mice received an intraperitoneal injection of 250 mg kg^−1^ of folic acid (F8758, Sigma; St. Louis, MO, USA).^[^ ^52^ ^]^ Mice were sacrificed 2, 7, or 14 days after surgery or injection.

For IFN‐α neutralization, C57BL/6 mice received an intravenous injection of 50 µg dose^−1^ anti‐IFN‐α mAb (22100, R&D system; Minneapolis, MN, USA) 30 min before UIRI, with an additional dose 3 days later. Mice treated with isotype IgG served as controls ^[^ ^55^ ^]^ and were sacrificed 7 days after surgery.

For adoptive transfer of macrophages, endogenous renal macrophages were depleted by intraperitoneal injection of clodronate liposomes (200 µL mouse^−1^) 1 h after UIRI surgery. After 24 h, 1 × 10⁶ BMDMs from *Gsdmd^fl/fl^* or *Gsdmd^ΔLyz2^* mice were injected intravenously. This cycle was repeated every 3 days, and mice were sacrificed 7 days after surgery.

### Cell Culture and Treatment

Mouse kidney proximal tubular epithelial cells (TKPTS, CRL‐3361, RRID: CVCL_UJ13) and human 293T cells (293T, CRL‐11268, RRID: CVCL_1926) were from the American Type Culture Collection. TKPTS were cultured in Dulbecco's modified Eagle medium/Nutrient Mixture F‐12 (DMEM/F12, Cat #C11330500BT, Gibco, Thermo Fisher Scientific; Halethorpe, MD, USA) with 10% fetal bovine serum (FBS) (Cat #10099‐141C, Gibco). 293T cells were cultured in DMEM (Cat #C11995500BT, Gibco) with 10% FBS and 1% antibiotics. The primary bone marrow‐derived macrophages (BMDMs) were prepared as described.^[^ ^56^ ^]^ Briefly, bone marrow from male mice was cultured in DMEM with 10% FBS, 1% antibiotics, and m‐CSF (20 ng mL^−1^, CB34, NovoProtein; Suzhou, China) for 7 days. BMDMs were over 99% CD11b⁺ and F4/80⁺ by Fluorescence Activating Cell Sorter. For co‐culture, TKPTS were exposed to 1% O_2_, 5% CO_2_, and 94% N_2_ at 37 °C with LPS (1µg mL^−1^, L2630, Sigma) for 24 h, then reoxygenated for 12 h to collect the conditional medium. BMDMs were treated with this conditional medium for 6 h, then with nigericin (10 µmol L^−1^, tlrl‐nig, InvivoGen; Carlsbad, CA, USA) for 2 h. In some experiments, the conditional medium was pre‐incubated for 6 h with a neutralizing anti‐IFN‐α antibody (2 µg mL^−1^) prior to nigericin challenge; an isotype antibody served as the control.

### Pathological Staining

PAS (G1008, Servicebio; Wuhan, China), HE (G1005, Servicebio), and Masson's staining (G1006, Servicebio) were performed according to the manufacturer's instructions. Tubular injury was scored by lesion area: 0 (no damage), 1(0–25%), 2 (26–50%), 3 (51–75%), 4 (76–100%).^[^ ^57^ ^]^

### Immunoblot Assay

The kidney cortex was homogenized or cultured cells scratched and lysed in RIPA buffer (20–188, Merck Millipore; Billerica, MA, USA) containing protease and phosphatase inhibitor cocktail (5892791001 and 4906837001, Roche; Basel, Switzerland) for 20 min on ice, following by centrifuged at 12000 *g* for 15 min at 4 °C. Protein concentration was quantified using the Enhanced BCA Protein Assay Kit (P0009, Beyotime; Shanghai, China), followed by SDS‐PAGE separation and transfer to polyvinylidene difluoride membrane. The membrane was incubated with primary antibodies at 4 °C overnight, followed by horseradish peroxidase (HRP)‐conjugated secondary antibodies (1:2000, Boster; Wuhan, China) for 1 h. Band intensities were determined by the Image J software (NIH; Bethesda, MD). The primary antibodies were listed in Table S3 (Supporting Information).

### Immunohistochemistry and Immunofluorescence Staining

Kidney tissues were fixed in 4% paraformaldehyde, dehydrated, cleared, and embedded in paraffin, as previously described.^[^ ^52^ ^]^ Sections (4 µm) were deparaffinized, rehydrated, antigen‐retrieved, and serum‐blocked. Primary antibody incubation was conducted overnight at 4 °C, followed by HRP‐conjugated secondary antibody for 1 h. Signals were visualized using a DAB kit (K5007, Dako; Glostrup, Denmark) and quantified with the Image J software. Primary antibodies used: anti‐human GSDMD‐N (Dr. Feng Shao's lab, 1:200), anti‐mouse F4/80 (MCA497G, Bio‐Rad, 1:200; Hercules, CA, USA); anti‐mouse CD3 (M7254, Dako, 1:2); anti‐mouse IFN‐α (18013‐1, Proteintech, 1:200; Wuhan, Hubei, China). For Immunofluorescence staining, 4 µm sections were incubated with primary antibodies overnight, followed by HRP‐conjugated secondary antibody for 1 h. Nuclei were counterstained with DAPI, and the sections were sealed with Prolong Gold anti‐quench reagent (P36930, Thermo Fisher Scientific). Images were captured using a Zeiss fluorescence microscope (Carl Zeiss, Oberkochen, Germany) and quantified with the ImageJ software. Primary antibodies used: anti‐mouse Collagen I (ab34710, Abcam, 1:50; Cambridge, MA, USA); anti‐human GSDMD‐N (Dr. Feng Shao's lab, 1:200), anti‐human CD68 (14‐0688‐82, Invitrogen, 1:50).

### Flow Cytometry Analysis

The mice kidneys tissues were harvested after perfusion with cold PBS and minced in cold RPMI 1640 containing 2% FBS, then were digested in a 10 mL digestion medium (RPMI 1640, 2% FBS, 1mg mL^−1^ Collagenase type II, 0.5mg mL^−1^ Dispase; all Gibco) for 30 min at 37 °C, and stirred at 180–200 rpm. The tissues were neutralized with PBS containing 1% FBS, then filtered (70 µm), centrifuged, and resuspended in PBS. The single‐cell suspension was incubated with surface staining flow cytometry antibodies for 30 min at 4 °C in the dark. The cells were then treated with the fixation/permeabilization kit (00‐5523‐00, Thermo Fisher Scientific). Succeeding washing, cells were stained with intracellular antibodies for 1 h at room temperature in the dark. Cells were analyzed using an AttuneNxT acoustic focusing cytometer (Thermo Fisher Scientific), with data analysis in FlowJo software (Version 10; Treestar Inc., San Carlos, CA, USA). Pyroptosis was evaluated using the FAM‐FLICA Caspase 1 YVAD Assay Kit (98, ImmunoChemistry Technologies; California, CA, USA) according to the manufacturer's instructions. Antibodies were listed in Table S4 (Supporting Information).

### RNA Sequence Analysis

BMDMs treated with conditional medium or control medium (with or without nigericin) were lysed in TRIzol Reagent for RNA extraction, cDNA synthesis, and library preparation. Libraries were sequenced on the Novaseq X Plus instrument (Illumina, California, CA, USA) via Majorbio Biopharm Technology Co. (Shanghai, China). Data analysis was on the Majorbio Cloud Platform (https://www.majorbio.com). PCA analysis assessed data reproducibility. Differential expressed genes were selected using DESeq2 R package (|log2(fold change) | ≥1, *P* adjust < 0.05). Kyoto Encyclopedia of Genes and Genomes (KEGG) and Gene Set Enrichment Analysis (GSEA) analyses explored key genes and pathways in macrophage pyroptosis activation.

### RNA Extraction and Real‐Time Quantitative Reverse Transcription PCR (RT‐qPCR)

Total RNA from kidney tissues or cultured cells was extracted using TRIzol reagent (15596018, Thermo Fisher Scientific). cDNA was synthesized using HiScript II Q RT SuperMix (R223, Vazyme; Nanjing, China). Real‐time qPCR was performed on the QuantStudio 5 (Applied Biosystems, Foster City, CA, USA) using SYBR Green Premix Kit (Q711, Vazyme). Partial qPCR primers were listed in Table S5 (Supporting Information). *Ifn‐β* (QM03294S), *Ifn‐λ2* (QM08870S), and *Ifnlr1* (QM08662S) primers were purchased from Beyotime (Shanghai, China).

### Chromatin Immunoprecipitation Quantitative PCR (ChIP‐qPCR)

Protein‐DNA interactions were evaluated by ChIP‐qPCR using a commercial Kit (9003S, CST, Danvers, MA, USA) following the manufacturer's instructions. Briefly, cells were cross‐linked with 1% formaldehyde, and chromatin was sheared by micrococcal nuclease. Immunoprecipitation was carried out using anti‐STAT2 (72604, CST, 1:50), anti‐IRF9 (14167‐1, Proteintech, 1:50), or control IgG (2729, CST, 5 µg). After washing, elution, and reverse cross‐linking, the purified DNA was analyzed by qPCR using the following primers: Stat2‐F 5′‐GTCCTGGCACCCACATAT‐3′, Stat2‐R 5′‐GGCTATCAATGGTGAAGTGT‐3′; Irf9‐F 5′‐CTTCACCATTGATAGCCACA‐3′, Irf9‐R 5′‐AATAGCAGTAAAG‐CAAACCC‐3′.

### RNA Interference

BMDMS matured over 7 days, and were transfected with *Jak2*, *Stat2*, *Irf9*, and *Ifnar1* small interfering RNA (siRNA) using Lipofectamine RNAiMAX (13778150, Thermo Fisher Scientific) according to the manufacturer's protocol for 48 h, followed by conditional medium treatment. siRNA sequences were listed in Table S6 (Supporting Information).

### Transfection and Luciferase Reporter Assay

The DNA fragments of the wild‐type and mutant mouse *GSDMD* promoter were cloned into the pMCS‐Fluc‐SV40‐hRluc‐Neo plasmid (Miaoling Biotechnology, Wuhan, China). pCMV‐*STAT1* (P44615), pCMV‐*STAT2* (P51197), and pCMV‐*IRF9* (P61294) plasmids were purchased from Miaoling Biotechnology. Validated constructs were transfected into 293T using Lipofectamine 3000 (L3000015, Thermo Fisher Scientific) according to the manufacturer's protocol. After 48 h, transcriptional activity was assayed using the Dual‐Luciferase Assay System (DL101, Vazyme).

### Serum Biochemical Analysis

Mouse blood was centrifuged at 3000 *g* for 25 min to obtain serum. Serum creatinine (SCr), blood urea nitrogen (BUN), aspartate aminotransferase (AST), alanine aminotransferase (ALT), and albumin were tested using an automatic biochemical analyzer (Cobas C 311, Roche; Basel, Switzerland).

### ELISA

Frozen kidney tissues (10mg) were homogenized in 200 µL PBS and centrifuged at 13000 *g* for 20 min at 4 °C; the supernatants were collected and stored at −80 °C. BMDMs' supernatants and TECs' conditional medium were centrifuged at 3000 *g* for 25 min and stored at −80 °C. IL‐1β (JL18442, J&L Biological; Shanghai, China) and IL‐18 (JL20253, J&L Biological), and IFN‐α (E‐EL‐M3054, Elabscience; Wuhan, China) levels were measured using commercial ELISA kits according to the manufacturer's instructions.

### Lactate Dehydrogenase (LDH) Assay

LDH release levels were measured using an LDH Cytotoxicity assay kit (C0017, Beyotime) per instructions.

### Propidium Iodide (PI) Staining

PI staining and Cell Plasma Membrane Staining with DiO (Green Fluorescence) were performed using the Beyotime kit (C1734, C1993) as per the protocol.

### Statistical Analysis

Data were presented as mean ± SEM and analyzed with GraphPad Prism software (Version 8.0; La Jolla, CA, USA). Unpaired Student's *t*‐test (2‐sided) was used for statistical analysis between two groups. One‐way ANOVA with Dunnett's or Bonferroni's test was used for multiple group comparison. Pearson correlation was used to calculate the correlation coefficient *r*. A *p*value of < 0.05 was considered statistically significant.

## Conflict of Interest

The authors declare no conflict of interest.

## Author Contributions

Y.P.X. and Y.T.W. contributed equally to this work. Y.P.X., Y.T.W., W.C., H.Y.L., and H.P.M. designed the study. Y.P.X., Y.T.W., S.M.J., Y.L., G.L.L., Y.C.L, and S.Y.L. performed the experiments. Y.P.X. and H.Y.L. analyzed the data. Y.P.X. and Y.T.W. wrote the article. Y.M.Z., Q.H.L., Y.Z., W.C., H.Y.L., and H.P.M. checked and revised the article. W.C., H.Y.L., and H.P.M. conceived and supervised the study. All authors read and approved the final manuscript.

## Supporting information



Supporting Information

Supplemental Movie 1

Supporting Information

Supporting Information

## Data Availability

The RNA sequencing data has been deposited in the National Center for Biotechnology Information (NCBI) Sequence Read Archive (SRA) database under BioProject accession number PRJNA1277127. The dataset is available at https://www.ncbi.nlm.nih.gov/bioproject/PRJNA1277127. The data supporting the results of this study are available from the corresponding author upon reasonable request.
