# Supplementary material for: Tubule‐Derived IFN‐α Promotes GSDMD‐Mediated Macrophage Pyroptosis to Drive Renal Inflammation and Fibrosis Through JAK2/STAT2 Activation
Source: Adv Sci (Weinh). 2025 Dec 12;13(11):e12278. doi: 10.1002/advs.202512278 (PMC12931230; doi:10.1002/advs.202512278)
Supplement: Supplementary file 1 — Supporting Information [file ADVS-13-e12278-s001.pdf]

Supporting Information

Tubule-Derived IFN-α Promotes GSDMD-Mediated Macrophage Pyroptosis to Drive Renal Inflammation and Fibrosis through JAK2/STAT2 Activation

Yiping Xu, Yating Wang, Siming Jiang, Yi Li, Guanglan Li, Yuchu Liu, Siyuan Li, Yiming Zhou, Qinghua Liu, Yi Zhou, Wei Chen, Hongyu Li*, Haiping Mao**

Table of contents

Supplementary Figure 1. Upregulation of *Gsdmd* mRNA level in UIRI- and FA-induced fibrotic kidneys.

Supplementary Figure 2. The gating strategy of B cells, T cells, neutrophils, and macrophages in the kidney of UIRI-induced mice in Figure 1G.

Supplementary Figure 3. Infiltrating macrophages exhibit elevated GSDMD expression and pyroptotic activity in fibrotic kidneys induced by UIRI and FA.

Supplementary Figure 4. Macrophage-specific deletion of *Gsdmd* has no effect on body weight or hepatic/renal function in healthy mice.

Supplementary Figure 5. Macrophage-specific deletion of *Gsdmd* does not ameliorate AKI.

Supplementary Figure 6. *Gsdmd* deletion attenuates $CCR2^-$ macrophage pyroptosis but does not alter macrophage polarization.

Supplementary Figure 7. Adoptive transfer of GSDMD-deficient macrophages alleviates renal inflammation and fibrosis.

Supplementary Figure 8. The injured TECs upregulate the expression of Interferon Stimulated Genes of the JAK-STAT pathway significantly.

Supplementary Figure 9. The correlations between *Stat1*, *Stat2*, and *Stat3* with *Gsdmd* in renal macrophages of UIRI mice.

Supplementary Figure 10. Expression of Type I/III IFNs in fibrotic kidneys and injured TECs, and correlation between *Ifnar1* and *Gsdmd* in renal macrophages.

Supplementary Movie.

Supplementary Table 1. Demographic and clinic data of CKD patients.

Supplementary Table 2. Bioinformatic prediction of STAT2 transcription factor binding sites within promoter regions of *Gsdmd* genes using the JASPAR database.

Supplementary Table 3. The list of primary antibodies used for the Immunoblot assay.

Supplementary Table 4. The list of antibodies used for Flow cytometry.

Supplementary Table 5. The list of primer sequences used for qPCR.

Supplementary Table 6. The list of siRNA sequences used in this study.

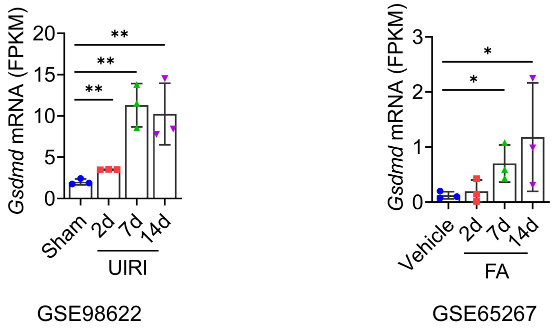

Figure S1. Upregulation of *Gsdmd* mRNA level in UIRI- and FA-induced fibrotic kidneys.

The *Gsdmd* mRNA expression in mouse kidneys after UIRI (GSE98622) and FA (GSE65267). *P < 0.05, **P < 0.01. Data are presented as mean ± SEM. Statistically significant differences were determined by one-way analysis of variance.

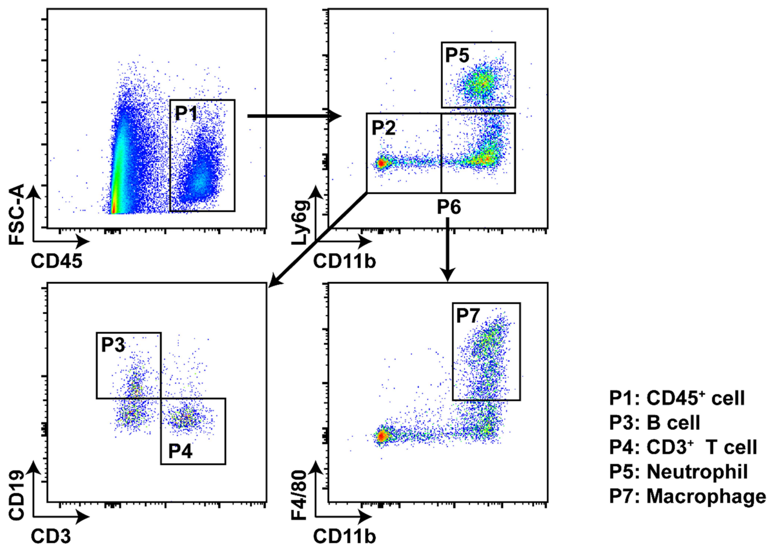

Figure S2. The gating strategy of B cells, T cells, neutrophils, and macrophages in the kidney of UIRI-induced mice in Fig. 1G.

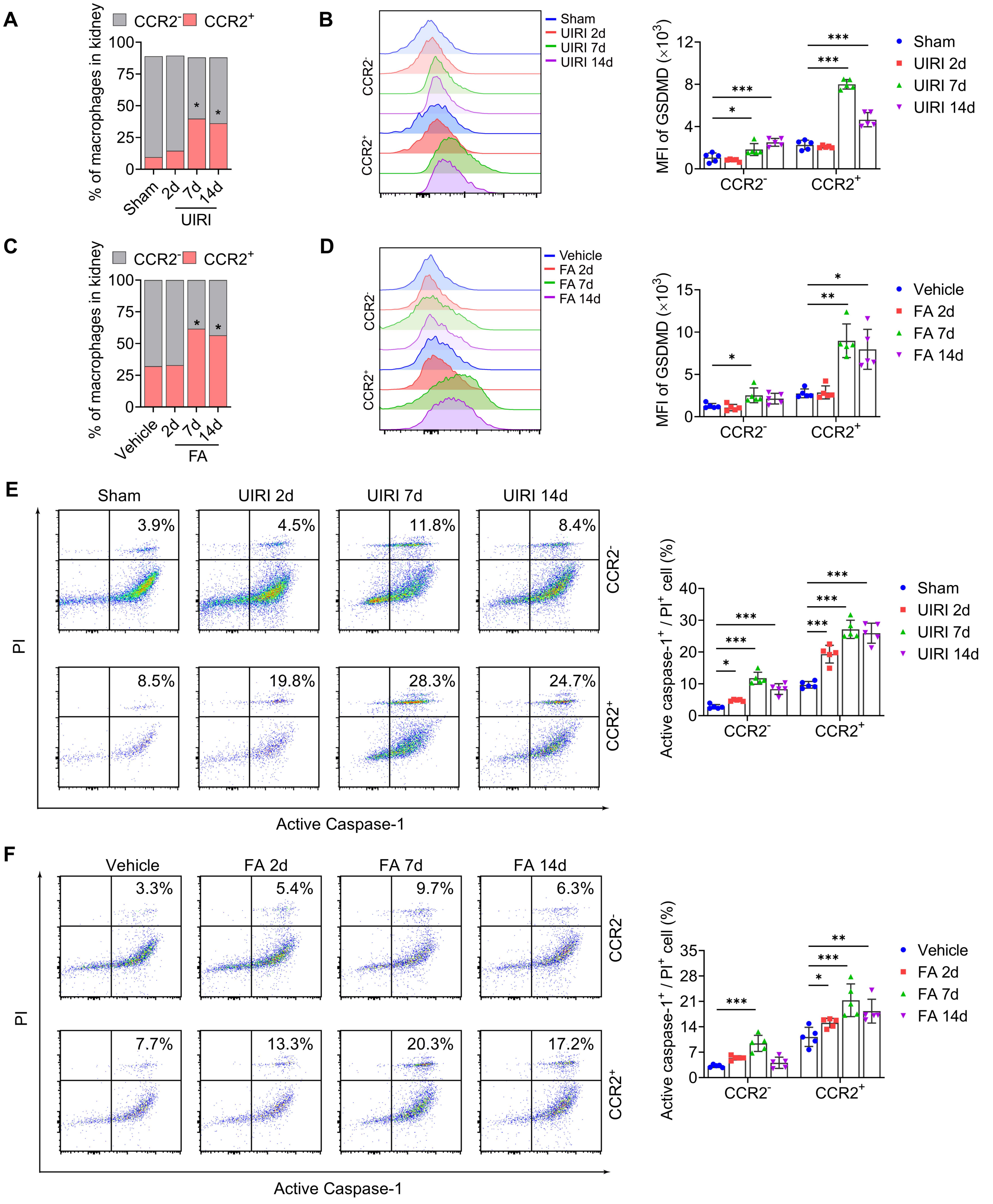

Figure S3. Infiltrating macrophages exhibit elevated GSDMD expression and pyroptotic activity in fibrotic kidneys induced by UIRI and FA.

(A-D) The cell proportions, the MFI, and quantitative data of GSDMD expression in CCR2+ and CCR2- macrophages from UIRI and FA-induced fibrotic kidneys (days 2, 7, and 14 after operation, n = 5 per group) or sham-operated/vehicle mice (n = 5 per group). **P* < 0.05 vs. Sham/Vehicle in A and C.

(E-F) The caspase-1+ PI+ cell proportions in CCR2+ and CCR2- macrophages from UIRI and FA-induced fibrotic kidneys (days 2, 7, and 14 after injection, n = 5 per group) or sham-operated/vehicle mice (n = 5 per group). **P* < 0.05, ***P* < 0.01, ****P* < 0.001. Data are presented as mean ± SEM. Statistically significant differences were determined by one-way ANOVA followed by Dunnett's test.

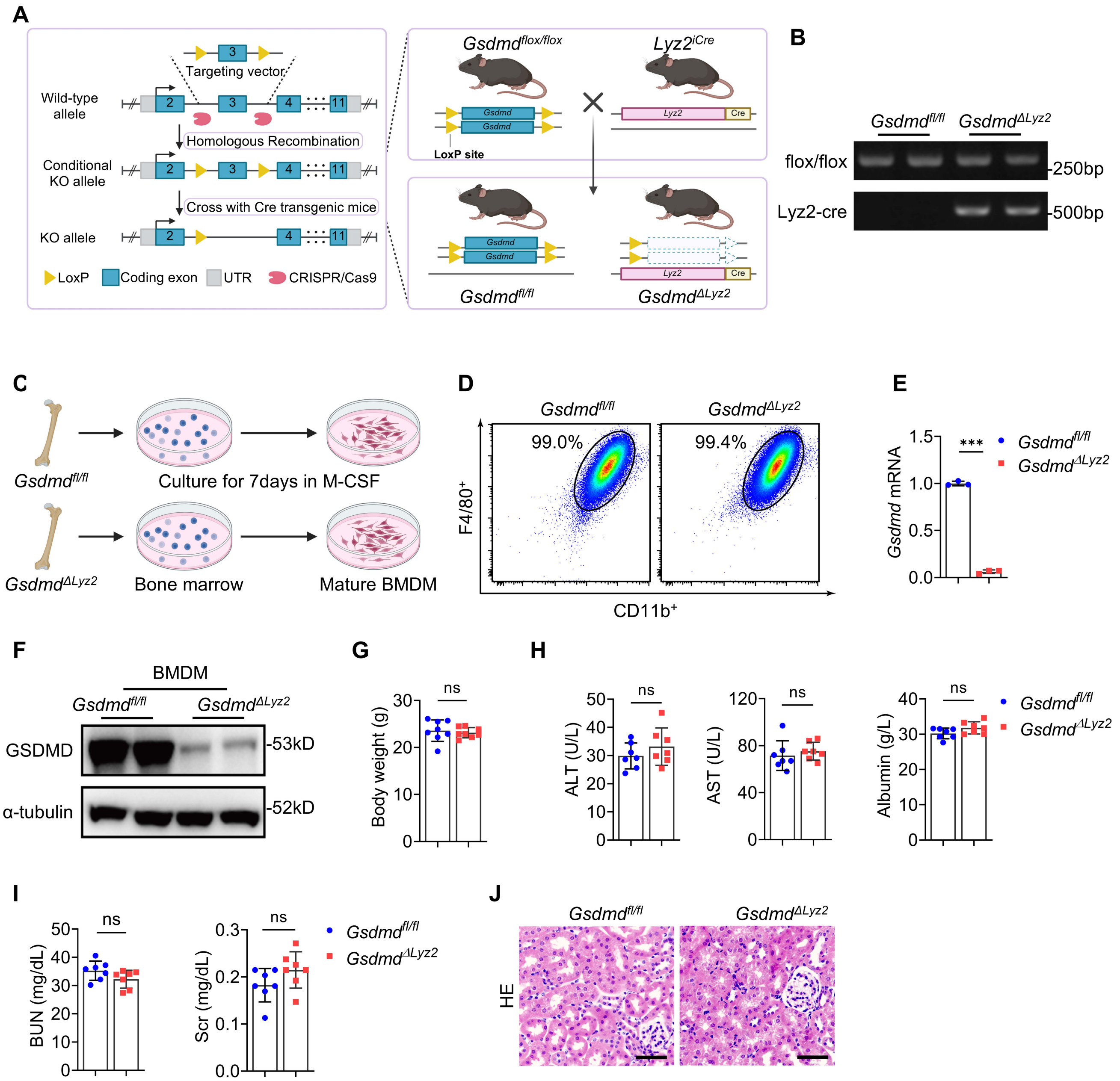

Figure S4. Macrophage-specific deletion of *Gsdmd* has no effect on body weight or hepatic/renal function in healthy mice.

(A) Schematic of macrophage-specific *Gsdmd* knockout in mice. Figure created with BioRender.com.

(B) Representative polymerase chain reaction (PCR) genotyping image of macrophage-specific *Gsdmd* deficiency mice.

(C) Schematic of bone marrow-derived macrophages (BMDMs) differentiation *in vitro*. Figure created with BioRender.com.

(D) Flow-cytometric analysis of BMDMs purity.

(E-F) Quantitative reverse transcriptase–polymerase chain reaction (qRT-PCR) and immunoblotting analyses of GSDMD in BMDMs from $Gsdmd^{fl/fl}$ or $Gsdmd^{\Delta Lyz2}$ mice (n = 3).

(G-I) Body weight, serum levels of alanine transaminase (ALT), aspartate transaminase (AST), albumin, blood urea nitrogen (BUN), and serum creatinine (Scr) of $Gsdmd^{fl/fl}$ and $Gsdmd^{\Delta Lyz2}$ mice under physiological conditions (n=6).

(J) Representative images of HE staining in kidney sections under physiological conditions. Bar = 50 μm. *P < 0.05, **P < 0.01, ***P < 0.001, ns: no significance. Data are presented as mean ± SEM. Statistically significant differences were determined by a 2-tailed Student's t-test.

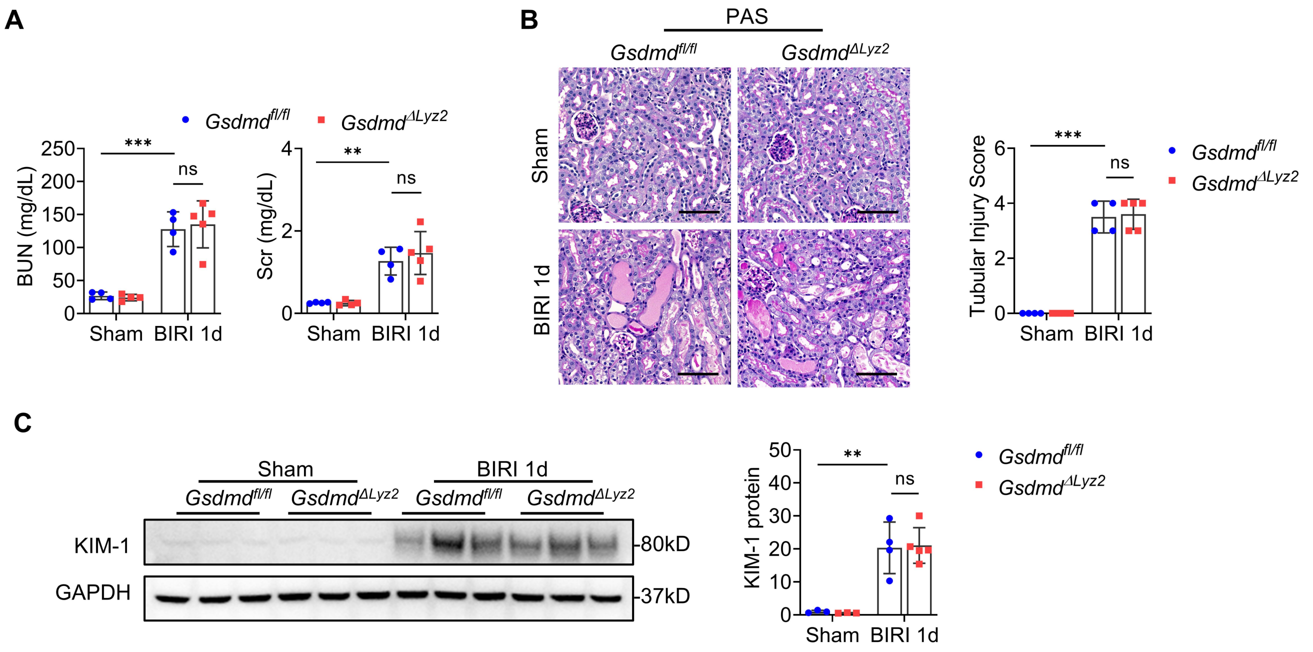

Figure S5. Macrophage-specific deletion of *Gsdmd* does not ameliorate AKI.

(A) Serum levels of blood urea nitrogen (BUN) and serum creatinine (Scr) of *Gsdmd*$^{fl/fl}$ and *Gsdmd*$^{\Delta Lyz2}$ mice at day 1 after BIRI (n = 4-5).

(B) Representative images and tubular injury scores of PAS staining in kidney sections at day 1 after BIRI (n = 4-5). Bar = 100 μm.

(C) Representative immunoblotting and quantitative data of kidney injury molecule-1 (KIM-1) protein level in the kidneys at day 1 after BIRI (n = 3-5). *$P < 0.05$, **$P < 0.01$, ***$P < 0.001$, ns: no significance. Data are presented as mean ± SEM. Statistically significant differences were determined by one-way ANOVA followed by Bonferroni's test.

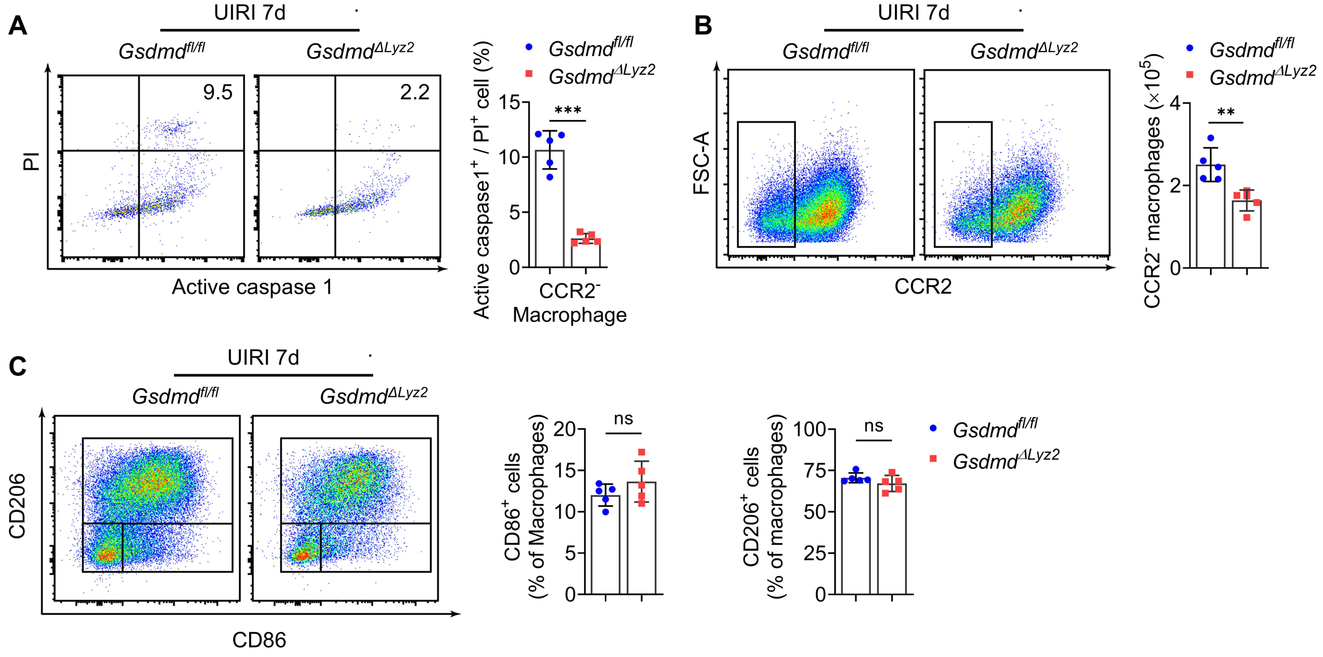

Figure S6. *Gsdmd* deletion attenuates CCR2⁻ macrophage pyroptosis but does not alter macrophage polarization.

(A) Flow-cytometric analysis of caspase-1$^+$ PI$^+$ cells among CCR2$^-$ macrophages in the kidneys at day 7 after UIRI (n = 5).

(B) Flow-cytometric analysis of CCR2$^-$ macrophages in the kidneys at day 7 after UIRI (n = 5).

(C) Flow-cytometric analysis of CD86$^+$ M1 and CD206$^+$ M2 macrophages in the kidneys at day 7 after UIRI (n = 5).

**P* < 0.05, ***P* < 0.01, ****P* < 0.001, ns: no significance. Data are presented as mean ± SEM. Statistically significant differences were determined by a 2-tailed Student's t-test.

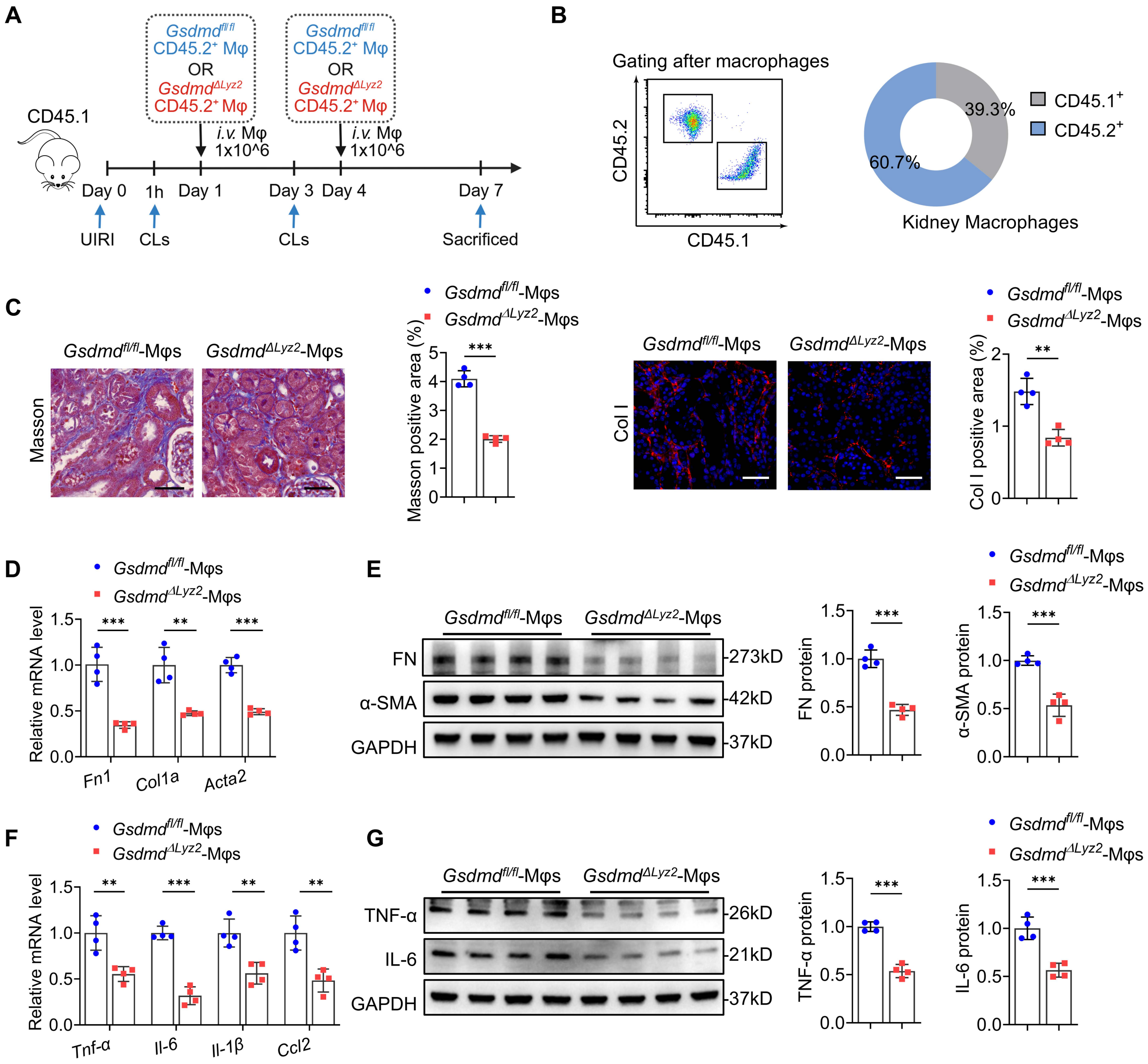

Figure S7. Adoptive transfer of GSDMD-deficient macrophages alleviates renal inflammation and fibrosis.

(A) Schematic of the experimental design; CD45.1⁺ mice received clodronate liposomes 1 hour after UIRI surgery to deplete endogenous macrophages, followed 24 hour later by adoptive transfer of BMDMs from either *Gsdmd*$^{fl/fl}$ or *Gsdmd*$^{\Delta Lyz2}$ CD45.2⁺ mice. The depletion-transfer cycle was repeated every 3 days. Mφ: macrophage.

(B) Percentage of CD45.1⁺ macrophages and CD45.2⁺ macrophages in the kidneys at day 7 after surgery (n = 5).

(C) Representative images and quantitative data of Masson's staining and collagen I (Col I) in kidney sections at day 7 after UIRI (n = 4). Bar = 50 μm.

(D) Quantitative reverse transcriptase–polymerase chain reaction (qRT-PCR) of the relative mRNA levels of renal *Fn1*, *Col1a*, and *Acta2* at day 7 after UIRI (n = 4).

(E) Representative immunoblotting and quantitative data of fibronectin (FN) and α-SMA protein levels in the kidneys at day 7 after UIRI (n = 4).

(F) qRT-PCR of the relative mRNA levels of renal *Tnf-α*, *Il-6*, *Il-1β*, and *Ccl2* at day 7 after UIRI (n = 4).

(G) Representative immunoblotting and quantitative data of TNF-α and IL-6 protein levels in the kidneys at day 7 after UIRI (n = 4). $*P < 0.05$, $**P < 0.01$, $***P < 0.001$, ns: no significance. Data are presented as mean ± SEM. Statistically significant differences were determined by a 2-tailed Student's t-test.

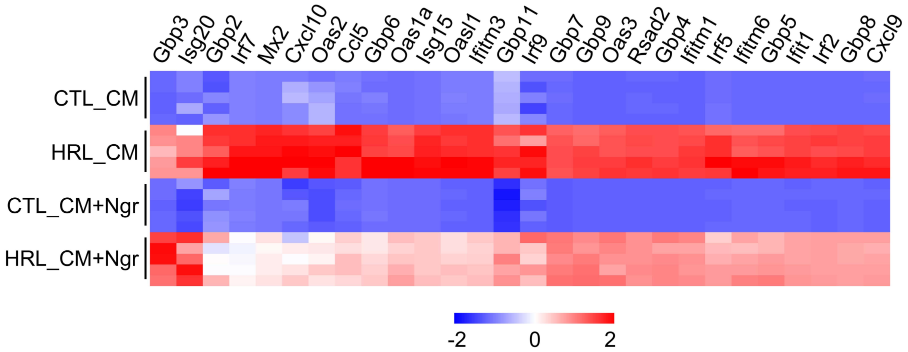

Figure S8. The injured TECs upregulate the expression of Interferon Stimulated Genes of the JAK-STAT pathway significantly.

Heatmap of canonical Interferon Stimulated Genes (ISGs) in BMDMs treated with HRL CM or CTL CM ± nigericin (n = 5).

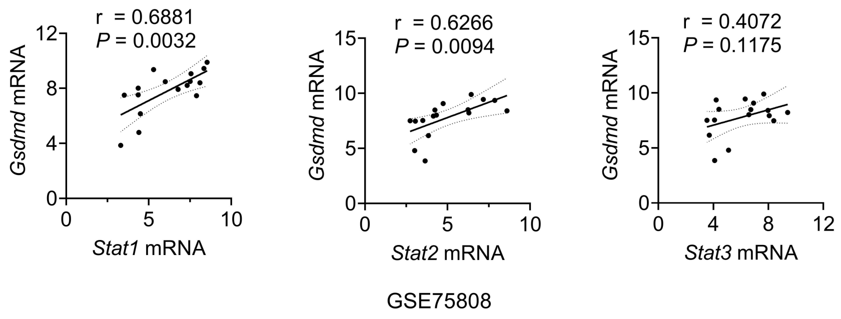

Figure S9. The correlations between *Stat1*, *Stat2,* and *Stat3* with *Gsdmd* in renal macrophages of UIRI mice. The correlation was analyzed using Pearson correlation. (n = 16, data from bulk RNA-Seq database GSE75808).

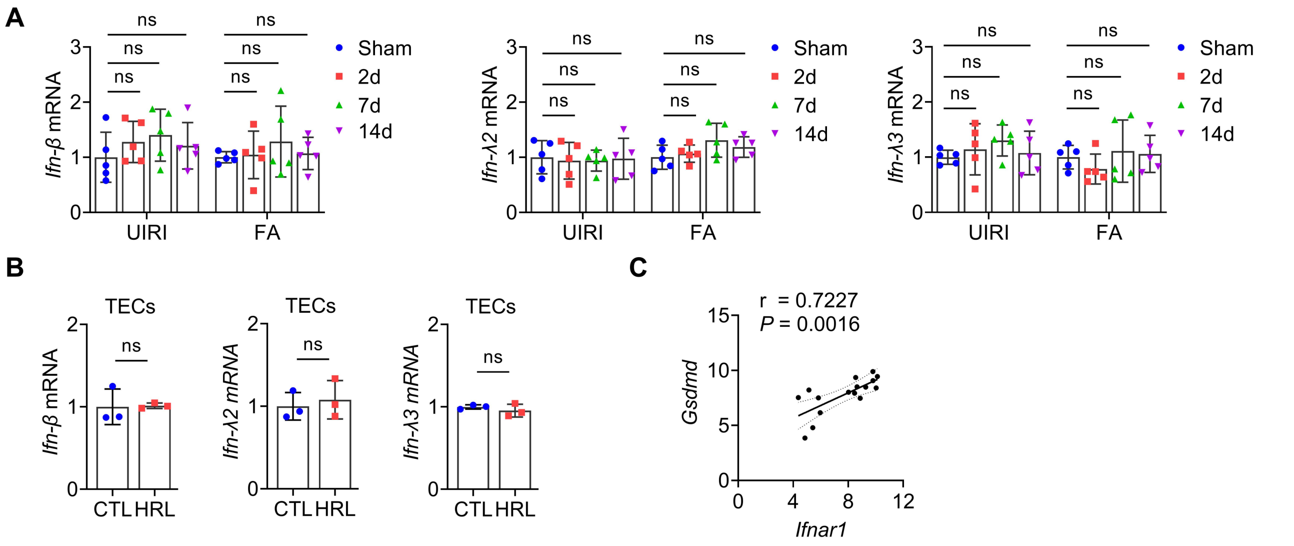

Figure S10. Expression of Type I/III IFNs in fibrotic kidneys and injured TECs, and correlation between *Ifnar1* and *Gsdmd* in renal macrophages.

(A) Quantitative reverse transcriptase–polymerase chain reaction (qRT-PCR) of the relative mRNA levels of *Ifn-β*, *Ifn-λ2*, and *Ifn-λ3* in the kidneys of UIRI and FA mice (n = 5). Statistically significant differences were determined by one-way ANOVA followed by Dunnett's test.

(B) qRT-PCR of the relative mRNA levels of *Ifn-β*, *Ifn-λ2*, and *Ifn-λ3* in TECs treated with hypoxia /reoxygenation (H/R) + LPS (HRL) or CTL (n = 3). Statistically significant differences were determined by a 2-tailed Student's t-test.

(C) The correlations between *Ifnar1* with *Gsdmd* in renal macrophages of UIRI mice. The correlation was analyzed using Pearson correlation. (n = 16, data from bulk RNA-Seq database GSE75808). Ns: no significance

Supplementary Movie

Time-lapse live-cell imaging of mouse BMDMs treated with HRL CM or CTL CM plus nigericin. Cells were cultured on collagen-coated glass-bottom dishes in serum-free DMEM, supplemented with Hoechst (blue) and propidium iodide (PI, red), to label nuclei and dead cells, respectively. Imaging was performed at 37 °C in a 5% CO_2 atmosphere using a Zeiss confocal microscope with a 40× objective. Images were acquired every 2 minutes for 1 hour and are displayed as DIC, Hoechst, and PI channels. Bar = 10 μm.

Table S1. Demographic and clinic data of CKD patients.

Characteristics	CKD patients (N=33)
**Age (year)**	
Mean±SEM	36±3
Range	18-70
**Gender-No. (%)**	
Male	19 (58)
Female	14 (42)
**SCr (mg/dl)**	2.15±0.29
**BUN (mg/dl)**	24.80±2.10
**eGFR (ml/min/1.73m2)-No. (%)**	
>90	6 (18)
60-89	7 (21)
30-59	9 (27)
15-29	5 (16)
<15	6 (18)
**Pathological diagnosis-No. (%)**	
IgAN	14 (42)
MN	7 (21)
FSGS	7 (21)
MCD	5 (16)

Table S2. Bioinformatic prediction of STAT2 transcription factor binding sites within promoter regions of *Gsdmd* genes using the JASPAR database.

Target Gene	Score	Relative score	Start	End	Predicted sequence
Gsdmd	11.6	88.2%	1613	1622	GAAATTGAAA
	10.3	85.6%	1453	1462	CAAACAGAAG

Table S3. The list of primary antibodies used for the Immunoblot assay.

Antibodies	Dilution	Source	Identifier
Anti-mouse GSDMD	1:1000	Abcam	Ab219800
Anti-mouse TNFα	1:1000	Abcam	Ab1793
Anti-mouse p-STAT2	1:1000	Abclonal	AP0284
Anti-mouse Caspase-1	1:1000	Adipogen	AG-20B-0042
Anti-mouse Collagen I	1:500	Boster	BA0325
Anti- mouse Fibronectin	1:1000	Boster	BA1772
Anti-mouse p-JAK2	1:1000	CST	3776
Anti-mouse JAK2	1:1000	CST	3230
Anti-mouse STAT2	1:1000	CST	72604
Anti-mouse p-STAT3	1:1000	CST	9145
Anti-mouse STAT3	1:1000	CST	9139
Anti-mouse IRF9	1:1000	CST	28845
Anti-mouse β-actin	1:1000	CST	4967
Anti-mouse GAPDH	1:1000	CST	5174
Anti-mouse α-Tubulin	1:1000	CST	12351
Anti-mouse KIM-1	1:1000	R&D systems	AF1817
Anti-mouse IFNAR1	1:1000	Santa	sc7391
Anti-mouse p-STAT1	1:1000	Selleck	F0451
Anti-mouse STAT1	1:1000	Selleck	F0263
Anti-mouse α-SMA	1:1000	Sigma	A5228
Anti-mouse IL-6	1:1000	Wanleibio	WL02841

Table S4. The list of antibodies used for Flow cytometry.

Antibodies	Clone	Source	Identifier
PE anti-mouse GSDMD	-	Abcam	Ab246713
APC-Cy7 anti-mouse CD45	30-F11	BD	557659
PE/Cyanine7 anti-mouse Ly-6G	1A8	Biolegend	127617
FITC anti-mouse CD3	17A2	Biolegend	100203
PE/Cyanine7 anti-mouse CCR2	SA203G11	Biolegend	150611
PE anti-mouse CD86	GL-1	Biolegend	105007
Brilliant Violet 421™ anti-mouse CD206	C068C2	Biolegend	141717
PE anti-mouse CD19	HIB19	Biolegend	302207
The fixable viability dye eFluor506	-	Invitrogen	65-0866-14
SB600 anti-mouse CD11b	M1/70	Invitrogen	63-0112-82
APC anti-mouse F4/80	BM8	Invitrogen	17-4801-82

Table S5. The list of primer sequences used for qPCR.

Gene	Species	Forward primer (5'to 3')	Reverse primer (5'to 3')
Gapdh	Mice	AGGTCGGTGTGAACGGATTTG	TGTAGACCATGTAGTTGAGGTCA
Actb	Mice	ACCCGCGAGCACAGCTTCTTTG	ACATGCCGGAGCCGTTGTCGAC
Gsdmd	Mice	GATCAAGGAGGTAAGCGGCA	AACACTCCGGTTCTGGTTCT
Jak2	Mice	GCGACGGGAACAAGATGTGA	AGAACATTGGCCTTCGCGG
Stat1	Mice	TGTCATCCCGCAGAGAGAAC	GAGCAGAGCTGAAACGACCTA
Stat2	Mice	GTTACACCAGGTCTACTCACAGA	TGGTCTTCAATCCAGGTAGCC
Stat3	Mice	CCCGTACCTGAAGACCAAGTT	AACGTGAGCGACTCAAACTG
Irf9	Mice	GTATGGTAAGGAGAAGGATGGC	TCGGAACTCTTGTTGAGGGC
Ifnar1	Mice	AGAGGTAGTCTCCAGCTCCG	GCTGCTCCACTTTAGGGTGT
Ifnar2	Mice	GCGTTAGGAAGAAGCACGAG	GGTGTGCATTTATTGGCAGG
Tnf-α	Mice	GAACTGGCAGAAGAGGCACT	AGGGTCTGGGCCATAGAACT
Il-1β	Mice	TCGTGAATGAGCAGACAG	AGAGGCAAGGAGGAAAAC
Il-6	Mice	CTGCAAGAGACTTCCATCCAG	AGTGGTATAGACAGGTCTGTTGG
Ccl2	Mice	AGGTCCCTGTCATGCTTCTG	TCTGGACCCATTCCTTCTTG
Ifn-α	Mice	AGGACTTTGGATTCCCGCAG	TCATTGAGCTGCTGGTGGAG
Ifn-λ3	Mice	CAAGGGTGCCATCGAGAAGA	AGAACACTGAGGAGGAGCCA
Fn1	Mice	ACAAGGTTCGGGAAGAGGTT	CCGTGTAAGGGTCAAAGCAT
Col1a	Mice	TGAACGTGGTGTACAAGGTC	CCATCTTTACCAGGAGAACCAT
Acta2	Mice	CTGACAGAGGCACCACTGAA	AGAGGCATAGAGGGACAGCA

Table S6. The list of siRNA sequences used in this study.

Gene	Species	Sense (5'to 3')	Anti-sense (5'to 3')
Jak2	Mice	UGGCAACAAGGAACAUAUUTT	AAUAUGUUCCUUGUUGCCATT
Stat2	Mice	CCAGAGACAGGGCUUAAUUTT	AAUUAAGCCCUGUCUCUGGTT
Irf9	Mice	UGCCAGCAGGAACCCUCCCTT	GGGAGGGUUCCUGCUGGCATT
Ifnar1	Mice	CAGAGACUACUUACUGUUUTT	AAACAGUAAGUAGUCUCUGTT
