# Supplementary material for: Tubule‐Derived IFN‐α Promotes GSDMD‐Mediated Macrophage Pyroptosis to Drive Renal Inflammation and Fibrosis Through JAK2/STAT2 Activation
Source: Adv Sci (Weinh). 2025 Dec 12;13(11):e12278. doi: 10.1002/advs.202512278 (PMC12931230; doi:10.1002/advs.202512278)

Fig. 1E

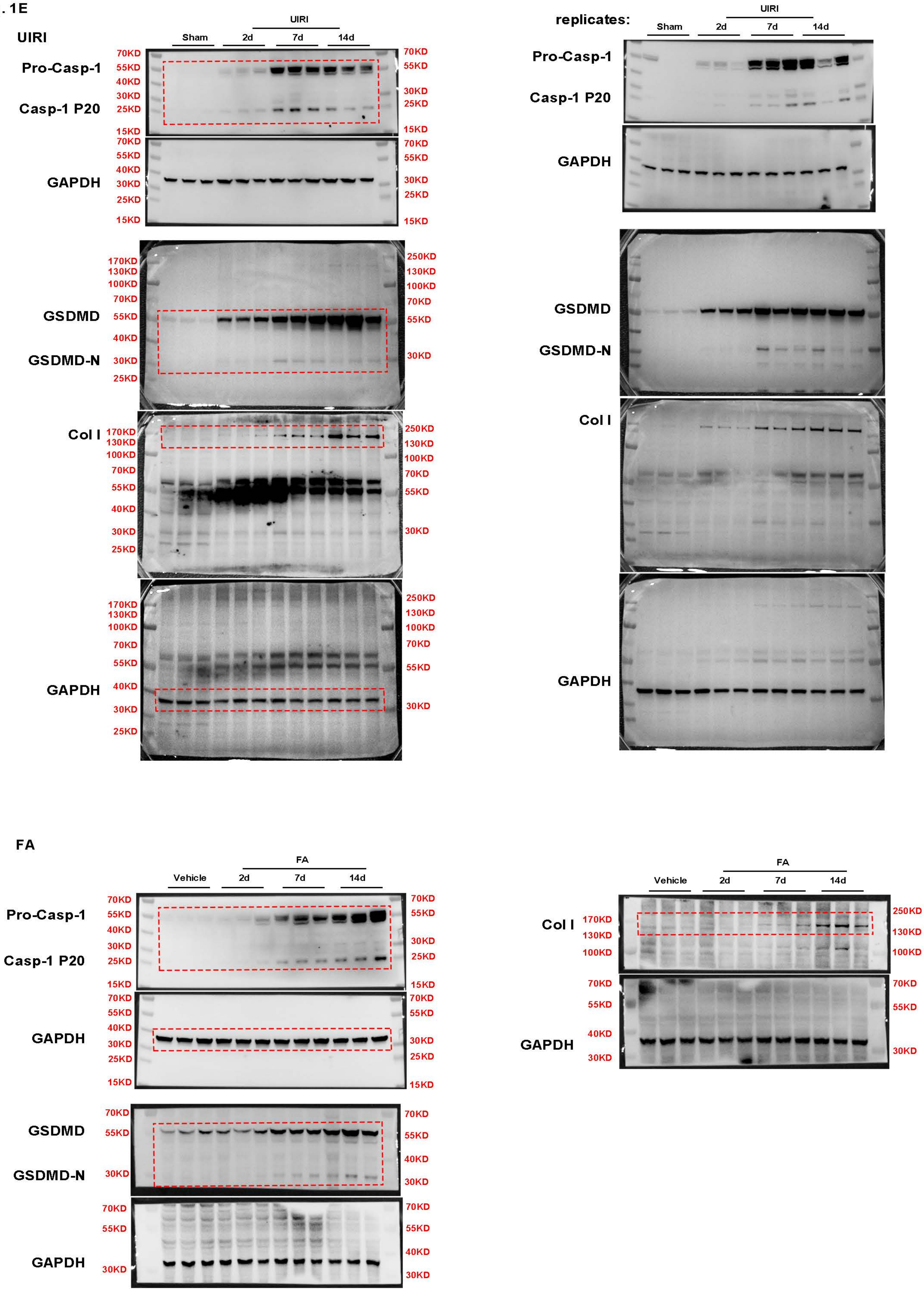

UIRI
Sham
2d
7d
14d
Pro-Casp-1
Casp-1 P20
GAPDH
GSDMD
GSDMD-N
Col I
replicates:
70KD
55KD
40KD
30KD
25KD
15KD
250KD
170KD
130KD
100KD
FA
Vehicle

Fig. 2B

UIRI

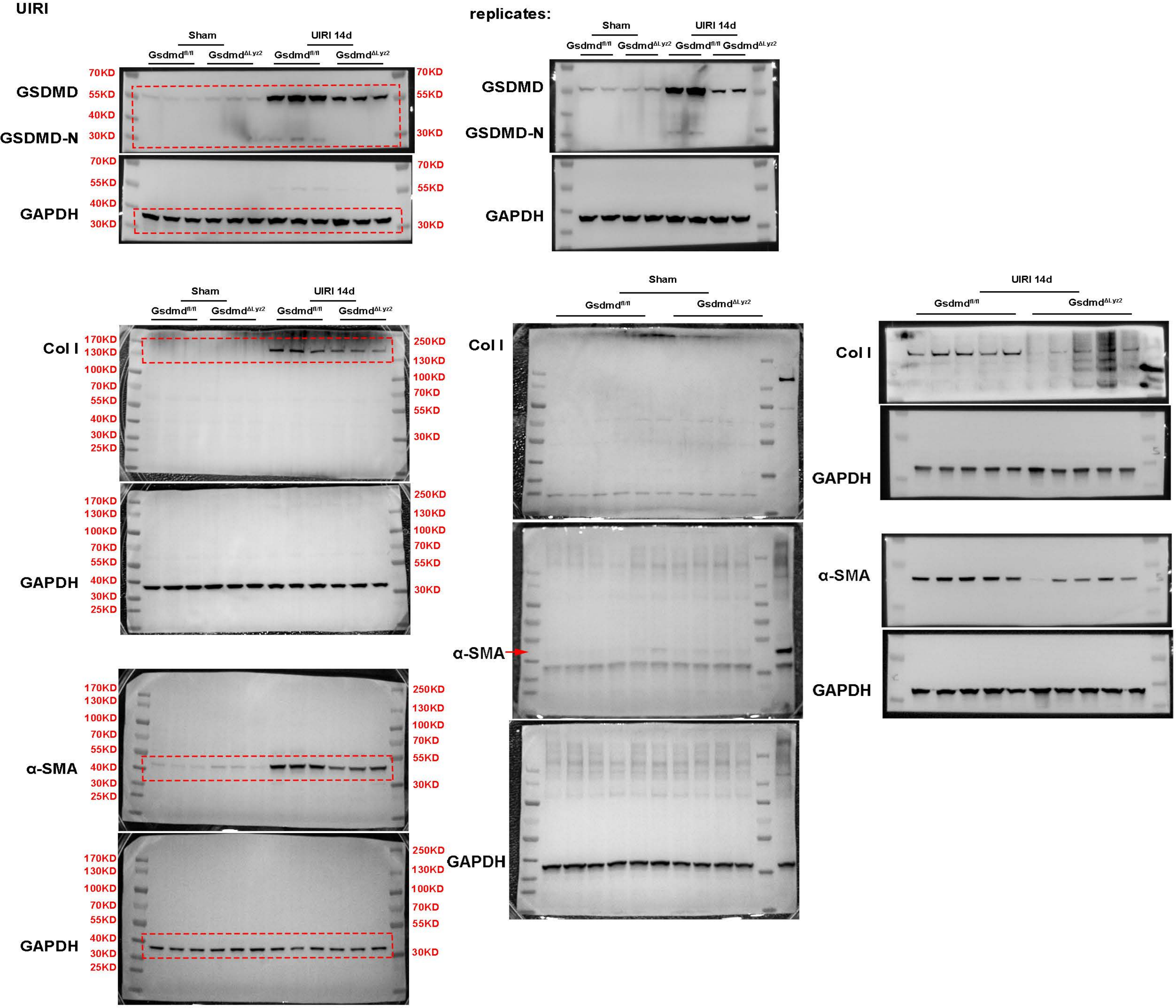
replicates:
Sham
UIRI 14d
Gsdmd$^{fl/fl}$
Gsdmd$^{\Delta Lyz2}$
GSDMD
GSDMD-N
GAPDH
Col I
α-SMA
70KD
55KD
40KD
30KD
250KD
170KD
130KD
100KD
25KD

Fig. 2D

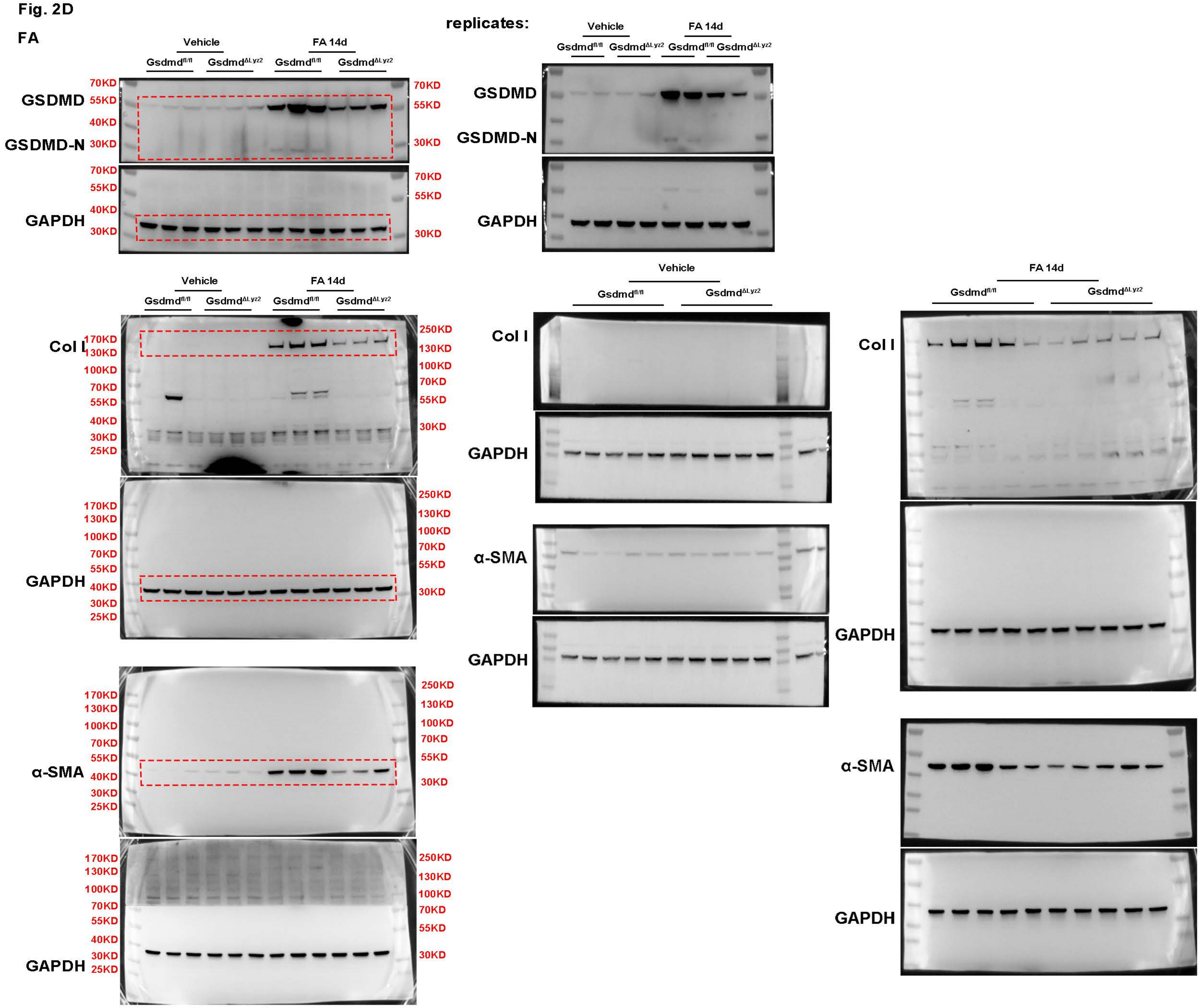
FA
replicates:
Vehicle
FA 14d
$Gsdmd^{fl/fl}$
$Gsdmd^{\Delta Lyz2}$
GSDMD
GSDMD-N
GAPDH
Col I
α-SMA
250KD
170KD
130KD
100KD
70KD
55KD
40KD
30KD
25KD

Fig. 3E

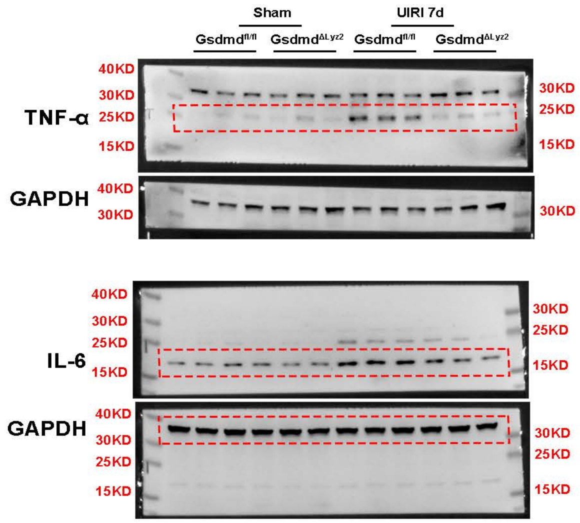

replicates:

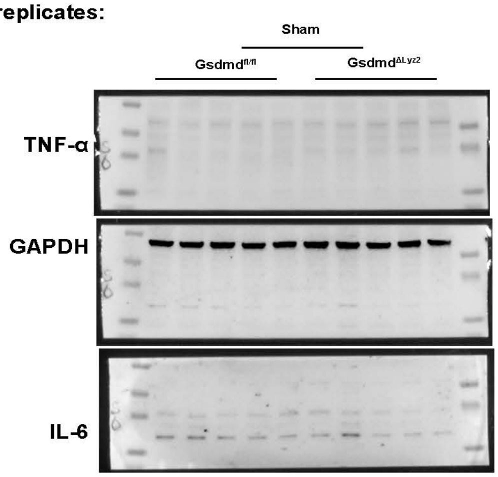

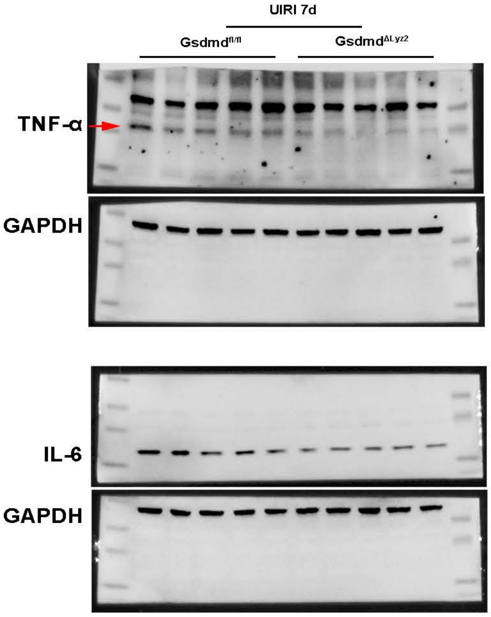

Fig. 4B

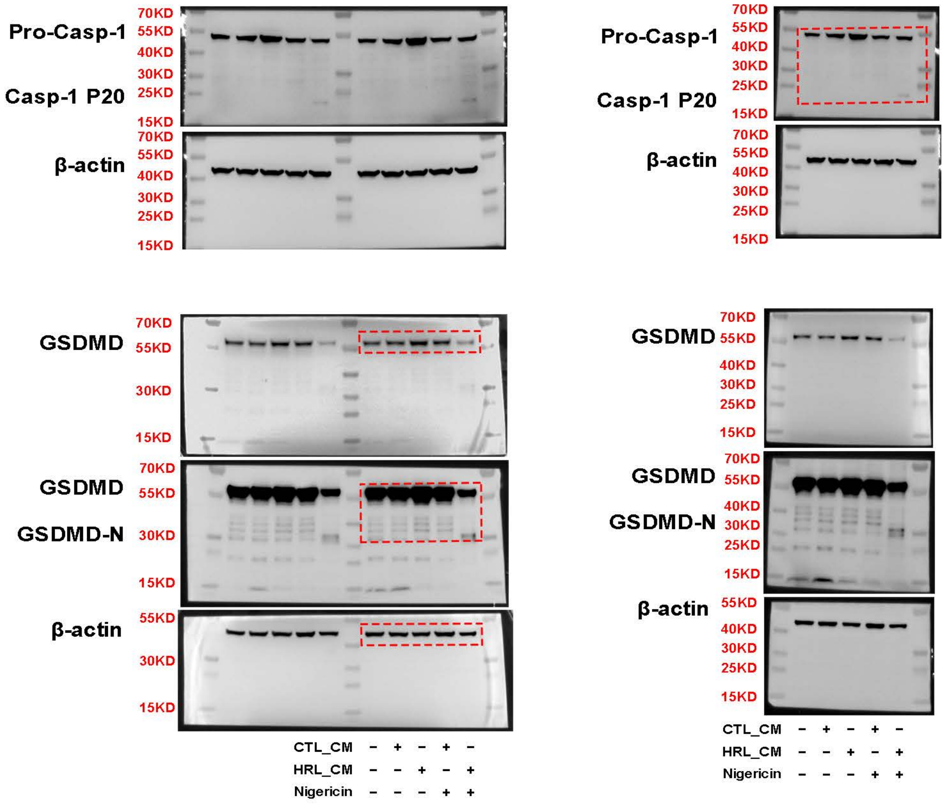
Pro-Casp-1
Casp-1 P20
β-actin
GSDMD
GSDMD
GSDMD-N
β-actin
70KD
55KD
40KD
30KD
25KD
15KD
CTL_CM – + – + –
HRL_CM – – + – +
Nigericin – – – + +

Fig. 5G

replicates:

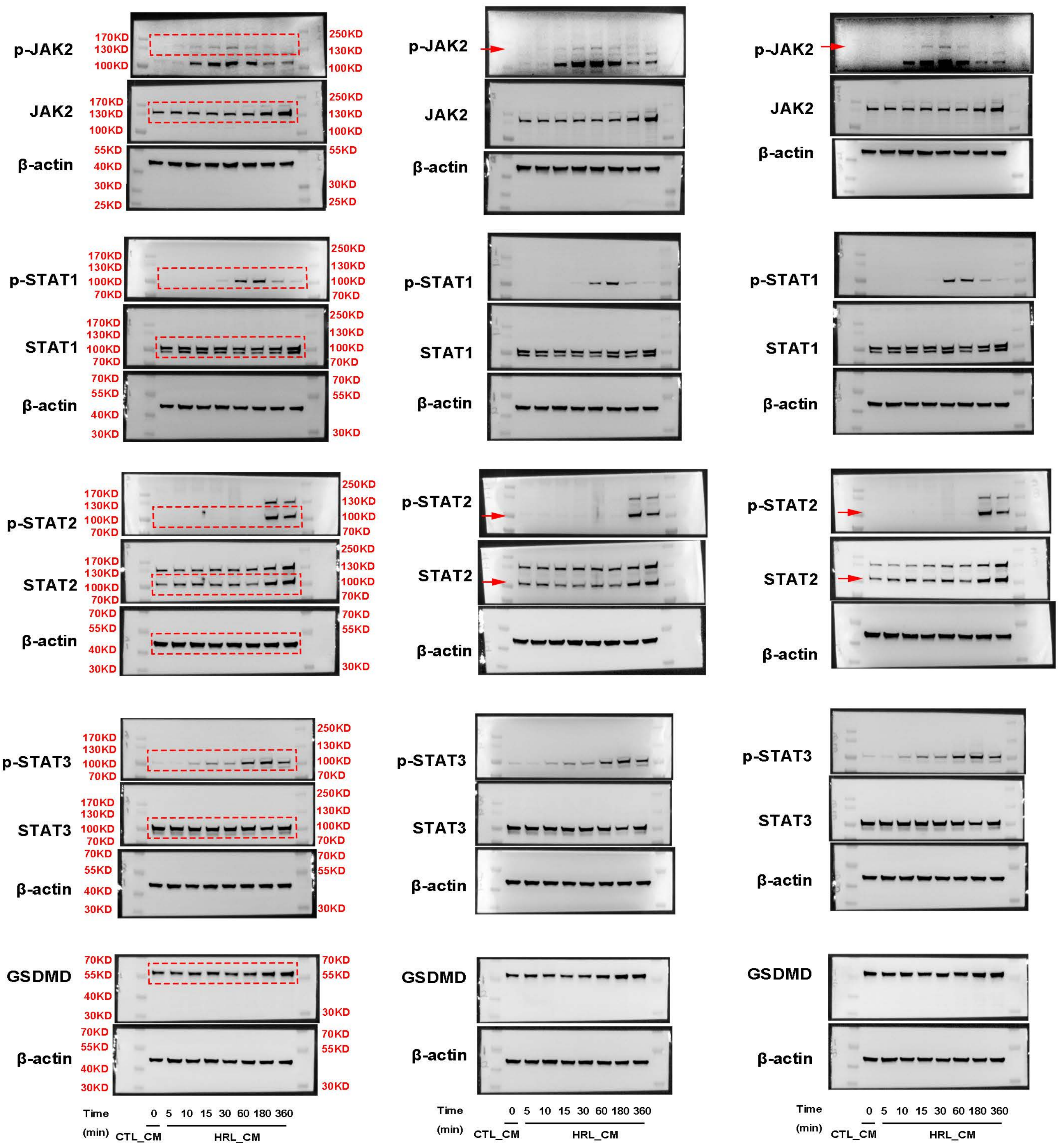

Fig. 5H

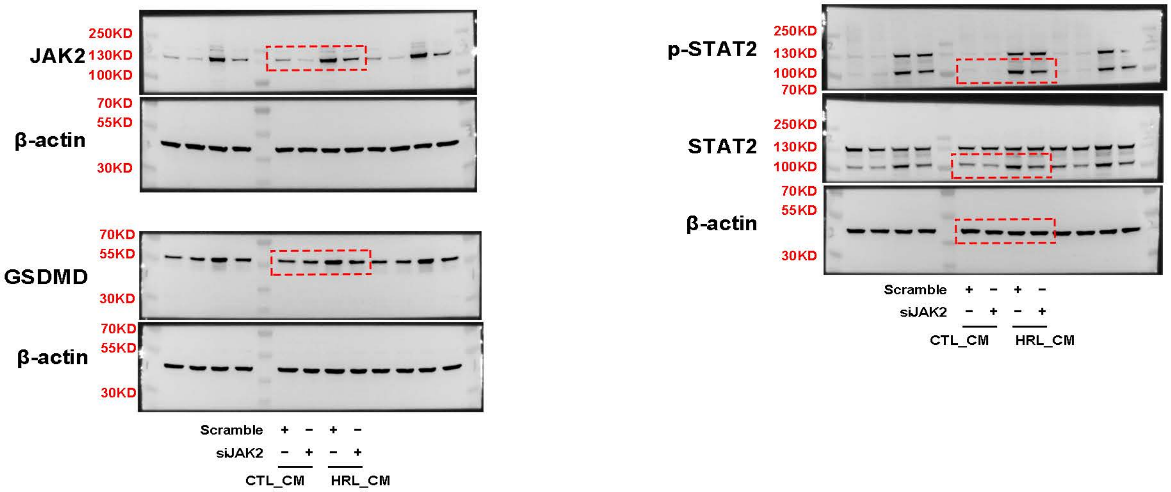
JAK2
250KD
130KD
100KD
70KD
55KD
β-actin
30KD
70KD
55KD
GSDMD
30KD
70KD
55KD
β-actin
30KD
Scramble + − + −
siJAK2 − + − +
CTL_CM
HRL_CM
p-STAT2
250KD
130KD
100KD
70KD
250KD
STAT2
130KD
100KD
70KD
55KD
β-actin
30KD
Scramble + − + −
siJAK2 − + − +
CTL_CM
HRL_CM

Fig. 5I

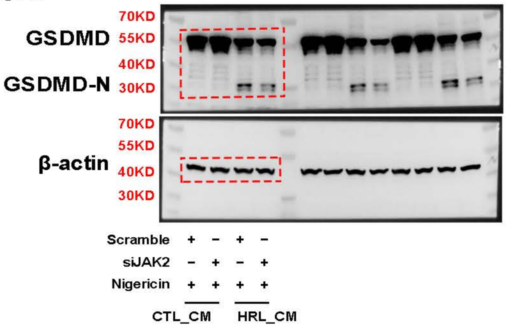
70KD
GSDMD
55KD
40KD
GSDMD-N
30KD
70KD
55KD
β-actin
40KD
30KD
Scramble + − + −
siJAK2 − + − +
Nigericin + + + +
CTL_CM
HRL_CM

Fig. 6H

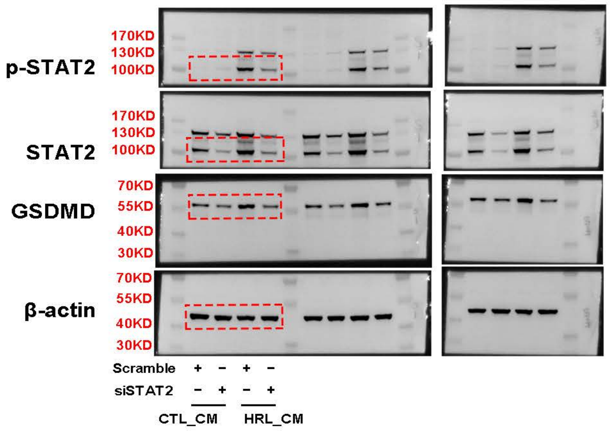

Fig. 6I

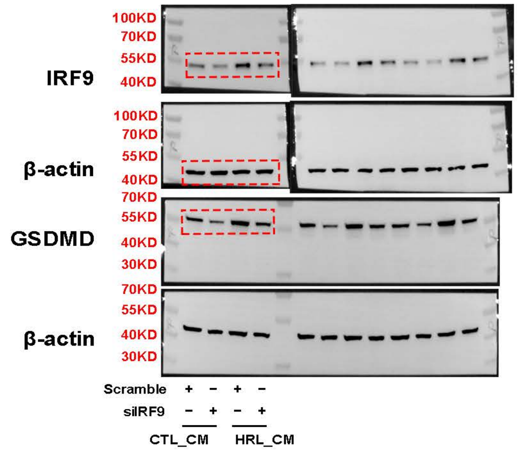

Fig. 6J

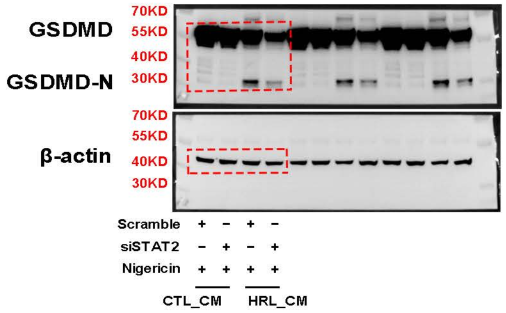

Fig. 6K

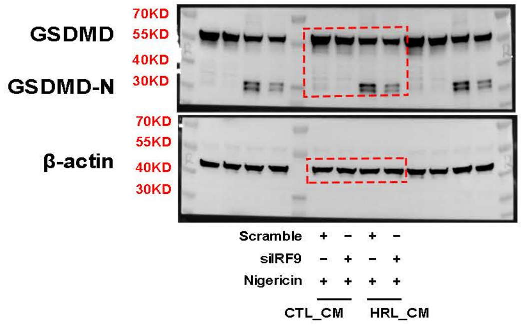

Fig. 7D

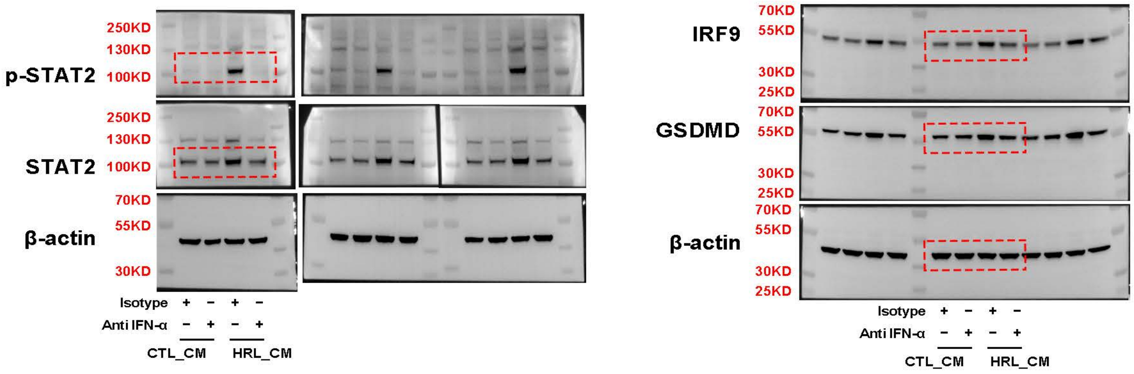

Fig. 7E

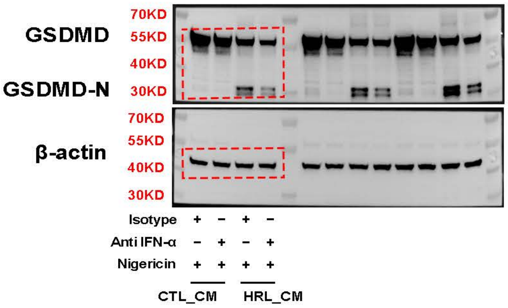

Fig. 7H

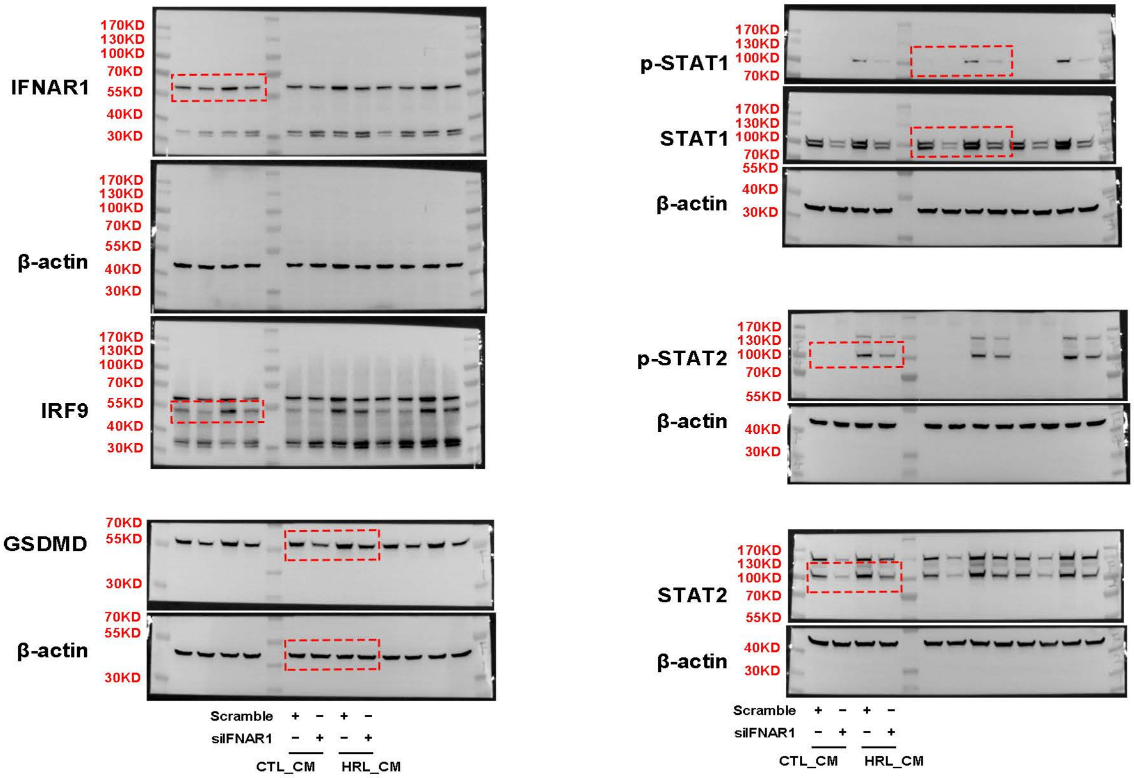

Fig. 7I

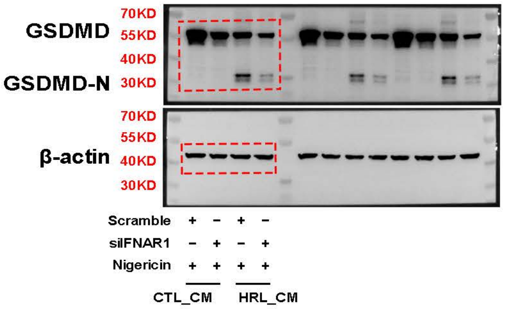

Fig. 8G

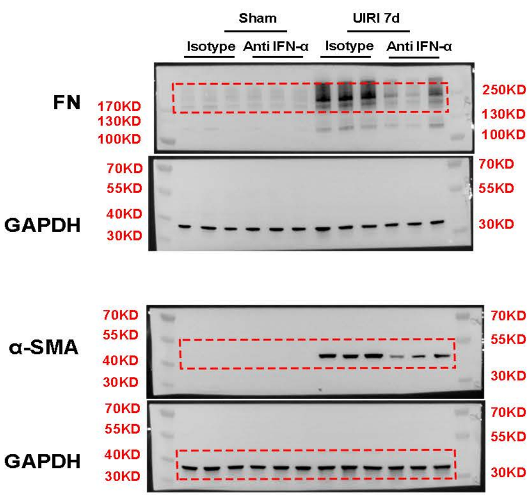

replicates:

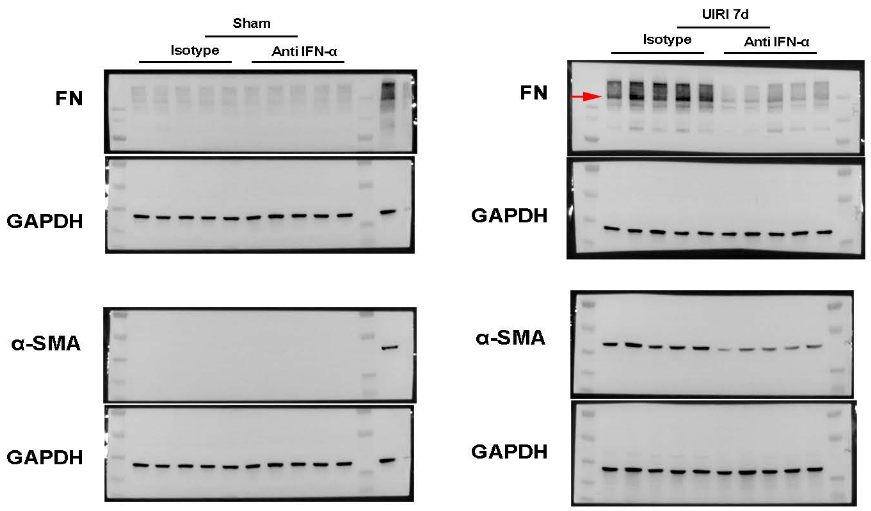

Fig. S4B

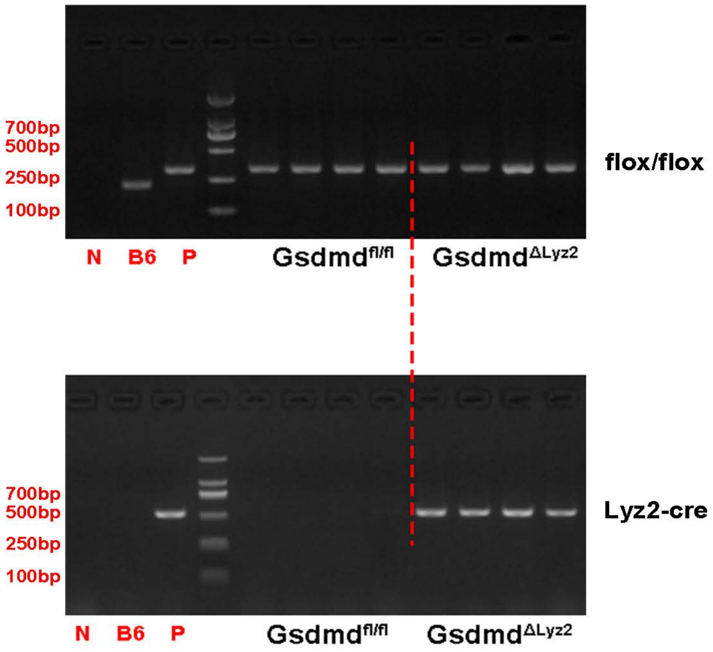

Fig. S4F

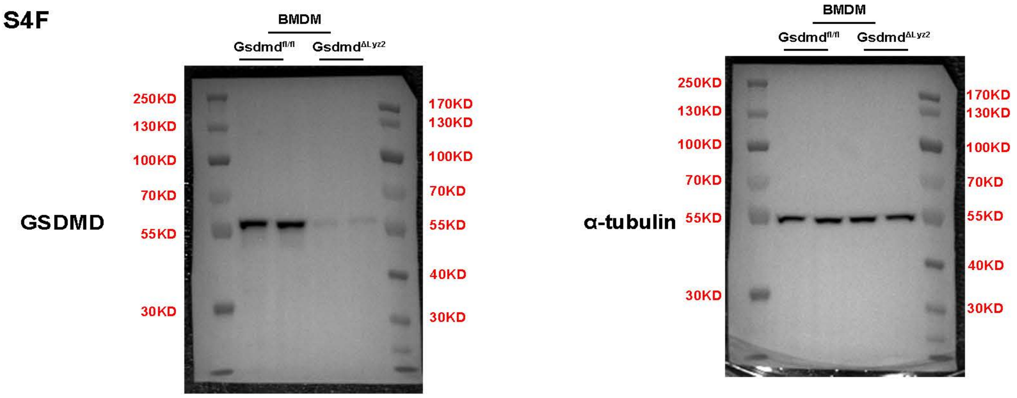

Fig. S5C

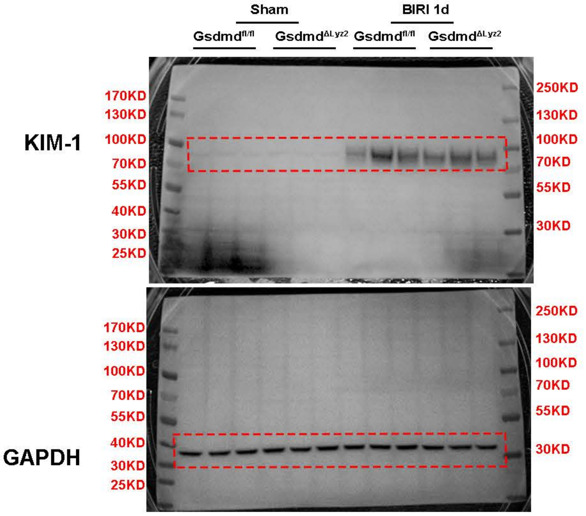

replicates:

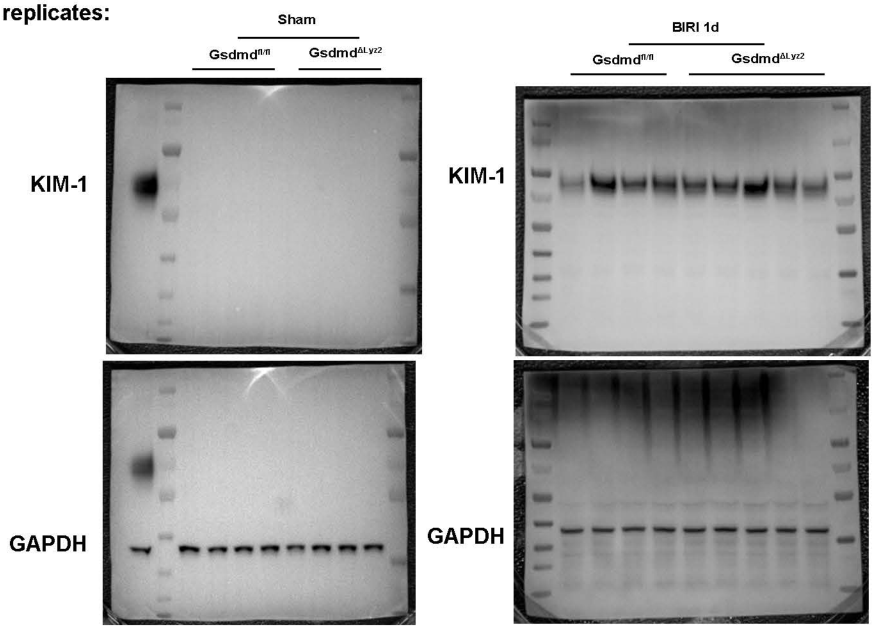

Fig. S7E

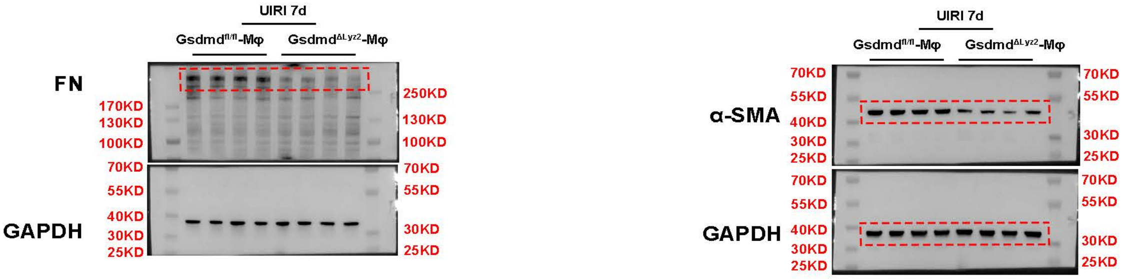

Fig. S7G

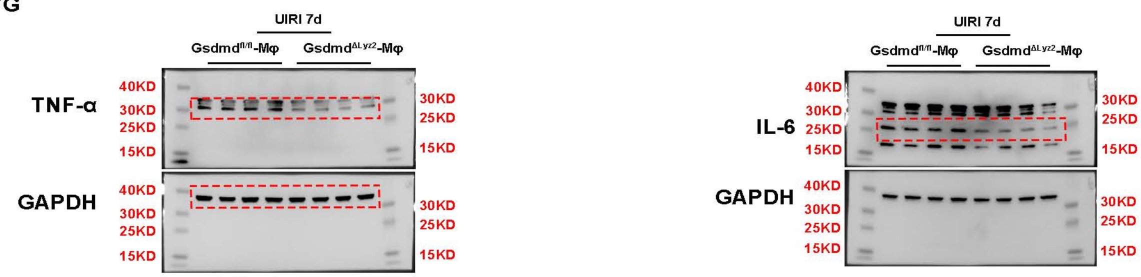

Supplement: Supplementary file 4 — Supporting Information [file ADVS-13-e12278-s002.pdf]
